# How impaired efficacy happened between Gancao and Yuanhua: Compounds, targets and pathways

**DOI:** 10.1038/s41598-017-03201-1

**Published:** 2017-06-19

**Authors:** Jin-gao Yu, Jianming Guo, Kevin Yue Zhu, Weiwei Tao, Yanyan Chen, Pei Liu, Yongqing Hua, Yuping Tang, Jin-ao Duan

**Affiliations:** 1 Jiangsu Collaborative Innovation Center of Chinese Medicinal Resources Industrialization, and National and Local Collaborative Engineering Center of Chinese Medicinal Resources Industrialization and Formulae Innovative Medicine, Nanjing University of Chinese Medicine, 138 Xianlin Road, Nanjing, 210023 Jiangsu Province China; 2 Jiangsu Key Laboratory for High Technology Research of TCM Formulae, Nanjing University of Chinese Medicine, Nanjing, 210023 China

## Abstract

As recorded in Traditional Chinese Medicine (TCM) theory, Gancao (Glycyrrhizae Radix et Rhizoma) could weaken the pharmacological effect or increase the toxicity of Yuanhua (Genkwa Flos). However, the theory has been suspected due to lack of evidence. Here, we investigate whether Gancao could weaken Yuanhua’s diuretic effect, if so, which chemicals and which targets may be involved. Results showed that Yuanhua exerted diuretic effect through down-regulating renal AQP 2, without electrolyte disturbances such as K^+^ loss which has been observed as side-effect of most diuretics. Gancao had no diuretic effect, but could impair Yuanhua’s diuretic effect through up-regulating renal AQP 2. Glycyrrhetinic acid (GRA) in Gancao could up-regulate AQP 2 and counteract the AQP 2 regulation effect of Yuanhuacine (YHC) and Ginkwanin (GKW) in Yuanhua. Network pharmacology method suggested that YHC, GKW and GRA could bind to MEK1/FGFR1 protein and influence ERK-MAPK pathway, which was verified by Western blotting. This study supports TCM theory and reminds that more attention should be paid to the safety and efficacy problems induced by improper combination between herbs. Moreover, we suggested that promising diuretics with less side effects can be developed from Chinese Medicines such as Yuanhua.

## Introduction

The safety and efficacy of traditional Chinese Medicine (TCM) have been concerned and questioned excessively for many years^1^, especially after the poisoning incidents of Longdanxiegan Pills, which was caused by Aristolochic acid in *Aristolochia manshuriensis* ^2, 3^. The safety and efficacy problems are still the main barriers in modern Chinese Medicine development. Today, with the modernization of the Chinese herbal formulas^4^, it is hypothesized that the safety or efficacy problems can be induced by combination of different types of herbs. As warned by the theory of “eighteen incompatible medicaments” (EIM), the most representative cases of improper herbal combination in TCM, certain herbs could interact with each other and cause impaired curative effect or enhanced toxicity^5^. However, this theory has not been well elucidated, the evidences and mechanisms still needs further explanation. Yuanhua (Genkwa Flos, YH) and Gancao (Glycyrrhizae Radix et Rhizoma, GC) are one of the herbal pairs, and the incompatibility between them has been questioned by some masters of TCM.

YH, the dried flower buds of *Daphne genkwa* Sieb. et Zucc. (Thymelaeaceae family), is a famous herbal in TCM since the age of Shennong’s Herbal Classics (Shen Nong Ben Cao Jing)^6^. It was described as a diuretic medicine, and was used to treat diseases caused by retained morbid fluid, such as hydrothorax, ascites, edema, asthma and arthritic^7–9^, although its diuretic activity still needs more detailed demonstration. Modern pharmacological researches have widely uncovered its anti-cancer^10, 11^, anti-inflammation^12^, and anti-oxidant^13^ activities. At least two types of chemical compounds in YH are believed to be responsible for its activities: flavonoids and Daphnane diterpene esters^8, 14, 15^.

GC, made of dried roots and rhizomes of *Glycyrrhiza uralensis* Fisch., *Glycyrrhiza inflata* Batalin, or *Glycyrrhiza glabra* L. (all from Leguminosae family), is the most frequently used herbal medicine in TCM, composing most Chinese herbal formulas^16^. GC can cause water retention if taken at high dose (above the upper limit of the Chinese Pharmacopeia). This effect may partially explain why GC is incompatible with YH. However, TCM doctors will not use GC at such high dose.

Since YH and GC have both been increasingly used or developed by doctors and scientists, there’s possibility that YH and GC could be used in combination. And more possibly, the chemical compounds of YH and GC could be mixed together by using other herbs, because chemicals in different herbs could be overlapped. Therefore, it’s also urgent to figure out the chemical essence of the incompatibility between YH and GC.

**Figure 1:**
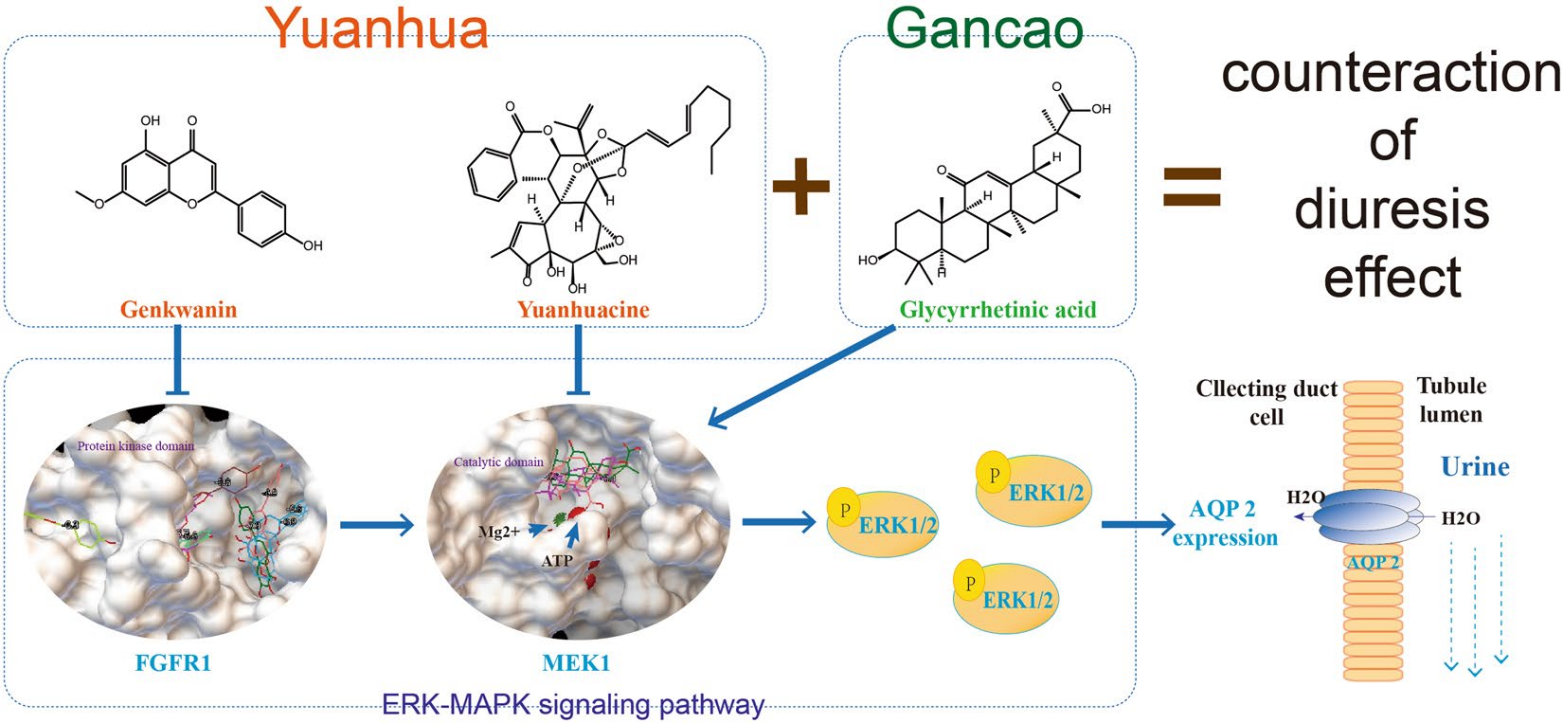
Research summary of this study. Our work began with evaluation of diuretic activity of Yuanhua (with or without combination of Gancao), and found that Gancao impaired the diuretic efficacy of Yuanhua. After studying some key factors in urine formation process, renal AQP 2 was proved the key target in Yuanhua’s diuretic activity and Gancao’s counteraction effect. Further, in renal collecting duct cells we found Yuanhuacine and Genkwanin dose-dependently decreased AQP 2 expression; oppositely, Glycyrrhetinic acid increased AQP 2 expression. The activity of these compounds were also verified *in-vivo*. Network pharmacology study revealed that these compounds could bind to MEK1 or FGFR1 protein and lead to regulations of ERK-MAPK pathway, which was verified by western blotting. At last, we conclude that the interaction in diuretic efficacy between Yuanhua and Gancao are due to differentially regulated renal AQP 2, which was mediated by ERK-MAPK signaling pathway.

In a word, this issue promoted us to investigate if GC could weaken YH’s diuretic efficacy under clinical doses, and if so, which chemical compounds could explain it, and what targets/pathways may be involved. The summary of this study has been showed in Fig. 1.

## Results

### YH increased urine excretion, GC did not increase urine excretion but impaired YH’s diuretic effect

To monitor urine excretion of mouse, mouse were loaded with normal saline (NS) and weighted 0 to 8 hours after water-loading, the accumulated weight loss was calculated as diuresis index (DI) to reflect urine output. The water-loading diuresis model made it possible to monitor urine output in a convenient way^17^, and the 8-hour procedure was proved suitable for evaluating diuretic effect of YH.

**Figure 2:**
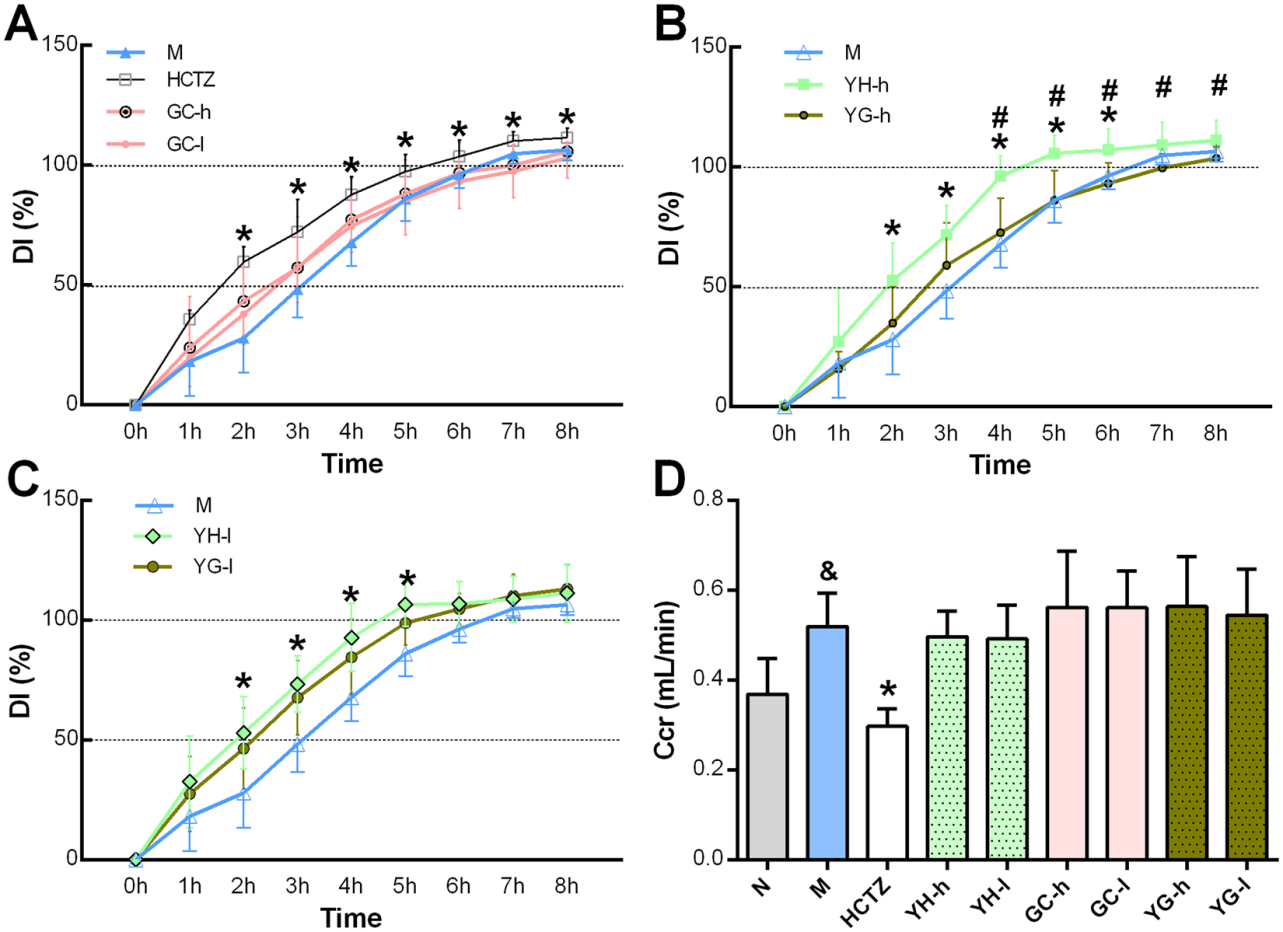
YH increased urine excretion and GC counteracted YH’s activity. (**A** to **C**) Accumulated urine output was calculated from animal weight loss 0 to 8 hours after water loading. The data were represented as diuresis index (DI), which means the percentage of weight loss in total water loading (n = 15). M group means the water loading model group. YG means combination of YH and GC at high or low dose. YH had diuretic activity at both high and low dose, however, when it combined with GC, the activity was counteracted. (**D**) The glomerular filtration rate (GFR) indicates primary urine amount, and can be calculated as plasma creatinine clearance rate (Ccr) (n = 8–10). Water loading can increase GFR, while HCTZ can decrease it. It’s found that YH’s diuretic effect and GC counteraction activity were not related to GFR. Other key factors in urine formation process are summarized in Supplementary Fig. S1. *P < 0.05 when HCTZ, YH-h and YH-l compared to M group; ^&^ *P* < 0.05 compared to N group; ^#^ *P* < 0.05 when YG-h group compared to YH-h group.

YH at high dose (YH-h) and low dose (YH-l) considerably increased DI between 2 to 6 hours, whereas hydrochlorothiazide (HCTZ) increased DI from 2 to 8 hours, which shows that they have diuretic effect. GC high dose (GC-h), GC low dose (GC-l), YH plus GC at high dose (YG-h) or low dose (YG-l) did not influence DI, which showed that although GC had no diuretic effect, but it can counteract the diuretic effect of YH (Fig. 2A–C).

### YH’s diuretic activity and GC’s counteraction effect were not related to glomerular filtration rate (GFR) but with urine concentrating ability

To elucidate the key process involved in YH’s diuretic effect and GC’s interference, we investigated some key factors involving in urine formation, which is summarized in Supplementary Fig. S1. Urine is generated from primary urine, and urine output is determined by total primary urine volume and urine concentrating ability. Total primary urine volume is determined by glomerular filtration rate (GFR), which is estimated by plasma creatinine clearance rate (Ccr) in clinical practice. Urine concentrating ability can be mainly reflected by urine osmotic pressure. So, the final amount of urine is determined by GFR and urine osmolality.

Water loading with NS (M group) can increase Ccr compared with normal group (N). HCTZ considerably reduced Ccr, which showed that HCTZ can decrease GFR (*P* < 0.01). While YH, GC and YG did not influence Ccr (Fig. 2D). These data suggests GFR is not responsible for YH’s diuretic activity or GC’s counteraction effect.

**Figure 3:**
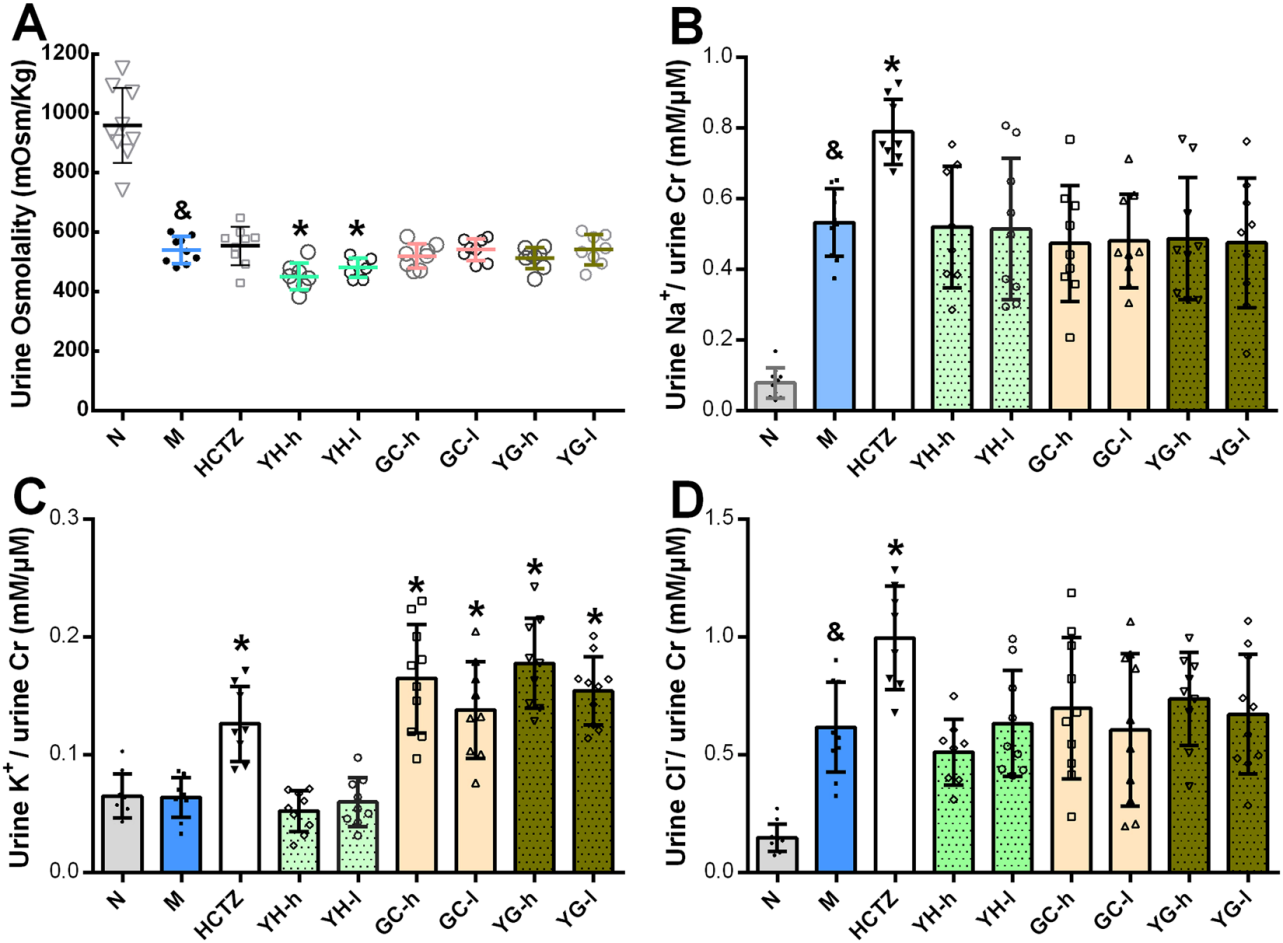
Urine output changes were related to urine concentrating ability and irrelevant to urine ion excretion. (**A**) Urine concentrating ability was reflected by urine osmolality (n = 7–10), higher osmolality means more concentrated primary urine. Water loading itself largely decreased urine osmolality, YH could further lower it, whereas GC and YG had no effect. HCTZ also failed to influence urine osmolality. (**B** to **D**) Total amount of Na^+^, K^+^, Cl^−^ excretion, calculated by urine electrolytes concentration standardized by urine Cr concentration (n = 7 to 10). Water loading with NS (M group) caused large amount of NaCl excretion, but have little influence on K^+^. HCTZ diluted urine through accelerating Na^+^ and Cl^−^ excretion, meanwhile caused K^+^ loss. GC also caused K+ loss while YH did not influence these ion excretions, so the UCF changes cannot be explained by ion excretion. ^&^ *P* < 0.05 compared to N group; *P < 0.05 compared to M group.

Urine osmolality shows the concentrating ability of the kidney (Fig. 3A), and in addition, urine concentrating fold (UCF) calculated by urine creatinine (Cr) and plasma Cr indicates how much water is reabsorbed from primary urine (Supplementary Fig. S2), lower urine osmolality or UCF means more diluted urine and less water reabsorption. Water loading largely diluted urine with low urine osmolality and low UCF. HCTZ treatment cannot influence urine osmolality due to its inhibition on electrolytes reabsorption, but can further lower UCF. YH reduced urine osmolality and UCF, but when GC was used in combination, this effect was abolished. GC cannot change urine concentrating ability under tested doses, however, it can still weaken YH’s effect. These data showed that urine concentrating ability was responsible for the diuretic activity of YH and counteraction effect of GC.

### YH’s diuretic effect and GC’s counteraction activity were not related to renal electrolytes reabsorption rate but AQP 2

Urine concentrating ability is mainly determined by two factors, urine electrolytes reabsorption and renal AQPs expression. Thereafter, we considered if the changes in urine concentrating ability was caused by urine electrolyte reabsorption variance. The three major ions Na^+^, K^+^, and Cl^−^ in urine were measured. Water loading with NS largely induced the excretion of Na^+^ and Cl^−^, but had no effect on K^+^. The positive drug HCTZ expectedly accelerated ion excretion of Na^+^, K^+^ and Cl^−^ (Fig. 3B–D)^18^, and disturbed the K^+^ ion homeostasis. YH had little influence on ion excretion, while GC led to K^+^ loss as HCTZ did, singly or combined with YH (Fig. 3C). The K^+^ loss has been reported by literature, because GC could exert “aldosterone-like” effect, due to increased cortisol level^19^. However, as for GC, the increased K^+^ excretion did not contribute to urine osmolality and there’s few report linking K^+^ loss and diuretic effect. We also measured plasma antidiuretic hormone (AVP) and aldosterone (ALD) level, the main two hormones regulating body water and electrolytes balance, again with negative results (Supplementary Fig. S3). These data showed that urine electrolyte reabsorption may not be involved in the diuretic effect of YH and counteraction effect of GC.

The AQP proteins are membrane proteins mediating high-efficiency cross-membrane water transport^20, 21^. They exist in every organ of mammalians for water metabolism, especially in the kidney^22, 23^. At least 8 kinds of AQPs exist in the epithelium cells of kidney tubules among which AQP 1, 2, 3, and 4 play the major role in the reabsorption of primary urine. Considering that more than 150 liters of primary urine is filtered by kidney glomeruli every day, and more than 99% of the water in urine is reabsorbed, slightly variation of AQPs can produce considerable changes on urine output^23, 24^. Among the AQPs, AQP 2 is the critical factor in water reabsorption in inner medullary collecting duct. AQP 2 expression can change quickly to adjust water balance, and it is activated by phosphorylation at Ser256 site (p256-AQP 2), and transported to the membrane. During the transportation, phosphorylation or dephosphorylation process at Ser261 (p261-AQP 2) and Ser269 sites (p269-AQP 2) are also essential according to literature^25^. AVP and its receptor AVPV2R play critical roles in AQP 2 regulation^26, 27^.

**Figure 4:**
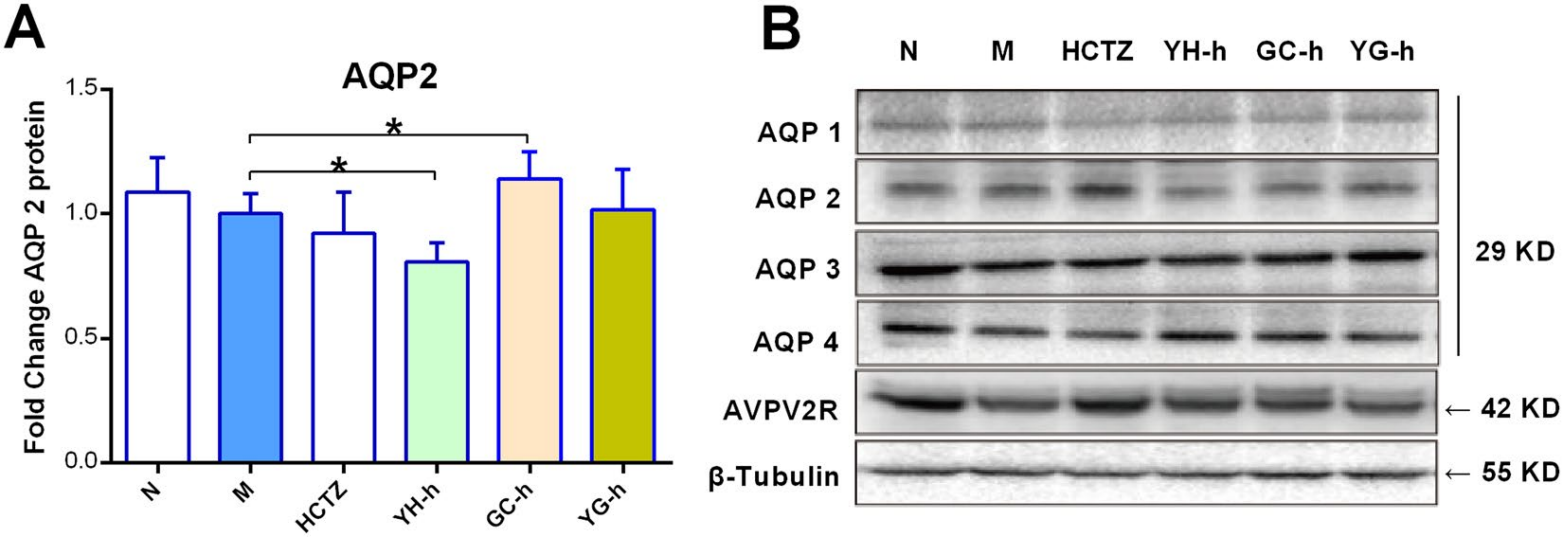
Urine output and urine concentrating ability changes were related to renal AQP 2 expression. Among renal AQPs and AVPV2R proteins, YH decreased AQP 2 expression while GC increased it. Other proteins were not considerably changed, which indicated that total renal AQP 2 may be the key target of YH and GC in diuretic activity. (**A**) Renal AQP 2 expression fold change, average number of M group is set as 1.0 (n = 6). *P < 0.05 as compared between the linked groups. (**B**) Blotting images of diuresis related proteins, including AQP 1, AQP 2, AQP 3, AQP 4 and AVPV2R. According to diuresis evaluation results, high doses of YH, GC and YG were selected for western blotting study, and one representative sample of each group is showed. The blots was cropped properly and the full length blots are presented in Supplementary Fig. S5.

According to DI curve, 4 hours after water loading is the best time point to further study the diuretic mechanism. Animals were sacrificed at this time point and kidneys of high dose groups were subjected to AQPs measurement by western blotting. As shown in Fig. 4, YH treatment can down-regulate AQP 2 protein expression, therefore suppress water reabsorption, leading to dilution of urine and increasing urine excretion. On the opposite side, GC treatment can up-regulate AQP 2, and when GC was used in combination with YH, GC abolished YH’s activity on AQP 2. Upstream AVPV2R protein, phosphorylation levels of AQP 2 or other AQPs were not influenced by YH or GC (Supplementary Fig. S4). It can be concluded that AQP 2 is the key factor through which YH exerts diuretic effect and GC counteracts the diuretic effect of YH.

### Yuanhuacine (YHC), Genkwanin (GKW) in YH and Glycyrrhetinic acid (GRA) in GC contribute to the AQP 2 regulation activity of YH and GC, respectively

The next step of this study is to investigate chemicals related with the AQP 2 regulation activity in GC and YH. The chemical compounds in YH and GC are complicated, it is hard to survey how many compound can regulate renal AQP 2. To simplify the situation, we select the most abundant chemicals that can be detected in blood after administered YH and GC, and use mouse renal collecting duct cells mIMCD3 to evaluated their activity on AQP 2 expression *in vitro*.

According to our previous report^28^, YHC is one of the most detectable diterpene-esters in animal plasma, and GKW, Apigenin (APG), Luteolin-5-*O*-glucoside (LUTG) are the most abundant flavones detectable in blood after oral administration of YH. GRA is considered the main active compound in GC, which is metabolized from Glycyrrhizic acid (GA) in the intestine before absorption after oral administration of GC^29^.

**Figure 5:**
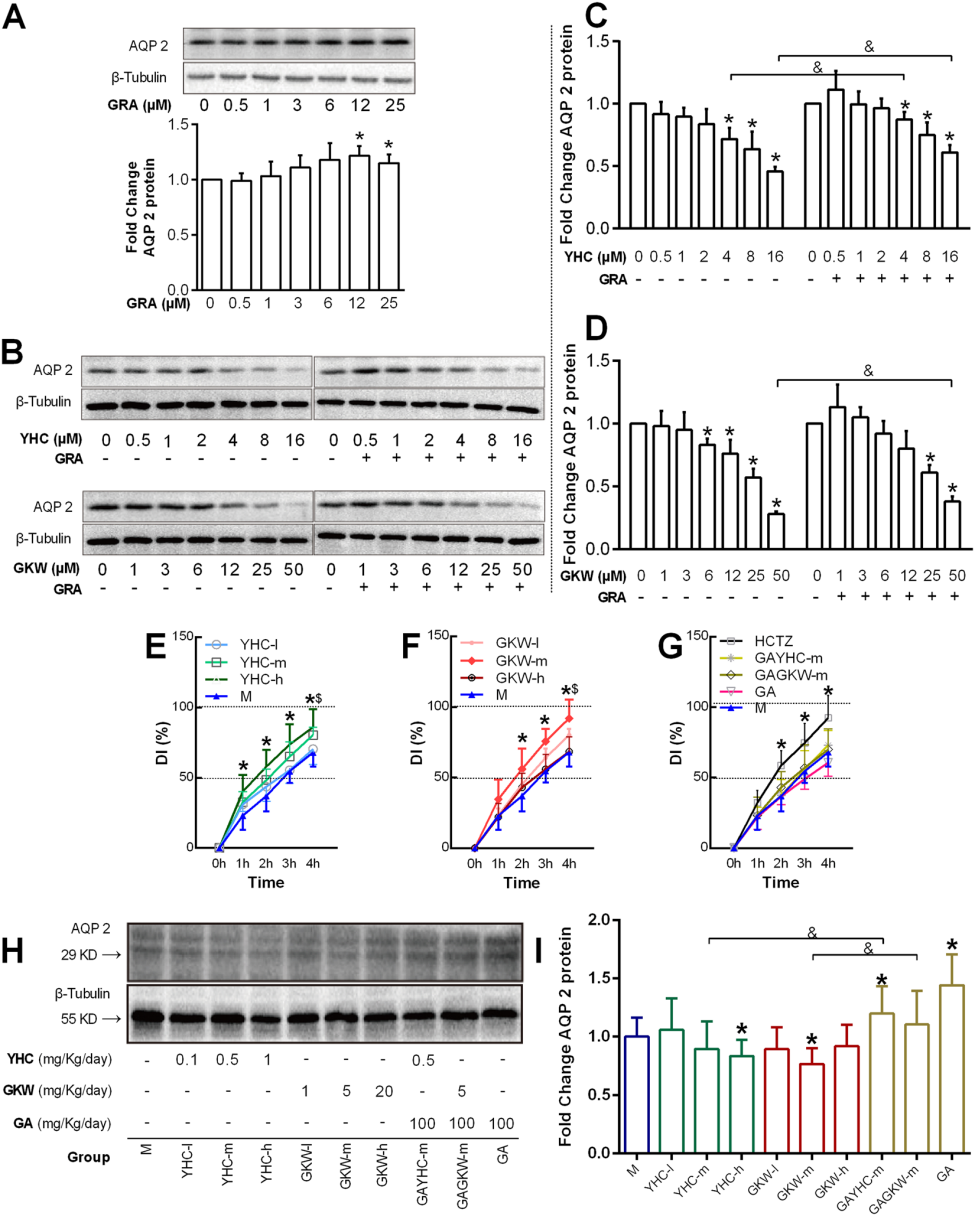
YHC, GKW and GRA (GA) are responsible for changes in AQP 2 regulation and diuresis effect *in vitro* and *in vivo*. The mIMCD-3 cells were planted in 6 well plate and cultured in RPMI1640 medium supplied with 10% fetal bovine serum. All doses were given during cell logarithmic phase, and the highest doses were restricted until cell proliferation was not affected. (**A**) Blotting image of AQP 2 expressed by mIMCD3 cells treated by GRA for 24 hours. Bar chart of fold changes is given, and concentrations are labeled below. (**B**) Blotting image of AQP 2 expressed by mIMCD3 cells treated by YHC, GKW singly or in combination with GRA, for 24 hours. When GRA was combined, the working concentration was 12 μM. The blots was cropped properly and the full length blots are presented in Supplementary Fig. S6. (**C** to **D**) Bar chart for AQP 2 expression with different treatment as with in (**B**) *P < 0.05 when compared with control group (none drug-treated), ^&^ *P* < 0.05 compared between groups with or without GRA treatment (n = 3). (**E** to **G**) Low, middle and high doses of YHC, GKW as well as special doses of HCTZ, GA and GA-YHC combination, GA-GKW combination were all submitted to diuresis mice model. Accumulated urine output was calculated from animal weight loss 0 to 4 hours after NS loading. The data were represented as DI. (**H** and **I**) representative blotting image and bar chart of renal total AQP 2 levels treated by different chemicals and their combination. Doses of each group are given below the image. *P < 0.05 when YHC-h or GKW-m groups compared with M group, ^$^ *P* < 0.05 when YHC-m or GKW-l compared with M group, ^&^ *P* < 0.05 when compared between groups with or without GA treatment (n = 6). The blots was cropped properly and the full length blots are presented in Supplementary Fig. S6.

Results showed that YHC and GKW but not APG or LUTG dose-dependently decreased total AQP 2 expression in mIMCD3 cells (Fig. 5B–D, Supplementary Fig. S7). In contrary, GRA considerably increased AQP 2 expression at 12 and 25 μM (Fig. 5A). Moreover, GRA attenuated the regulation effects of YHC and GKW on AQP 2.

Then, YHC and GKW were submitted to *in-vivo* study to confirm the diuresis activities of the two compounds as well as their regulation effects on renal total AQP 2 level. The designation basis of the doses were as follow: For YHC, since we have previously tested its toxicity on mice at 10 mg/Kg/day dose, so we designed 1, 0.5, and 0.1 mg/Kg/day dose in this study. And for GKW, in reference to the dose of HCTZ and Tolvaptan, 5 mg/Kg/day was used as the middle dose, and 20 and 1 mg/Kg/day were used as high/low doses respectively. GA was also used alone or combined with YHC and GKW, the dose was set at 100 mg/Kg/day according to literatures^19^. We choose GA rather than GRA because GA is the main form of triterpenoid saponin in GC, and it can be metabolized into the circulation form as GRA in the intestine. Results showed that YHC of 1 mg/Kg/day (YHC-h group) significantly promoted urine output from 1 to 4 hours after NS loading (*P* < 0.05); YHC of 0.5 mg/Kg/day (YHC-m group) only increased DI at the 4 hour point; and YHC of 0.1 mg/Kg/day (YHC-l group) did not change DI (Fig. 5E). As for GKW, 5 mg/Kg/day dose (GKW-m group) had the most strong effect, promoting urine output from 2 to 4 hours, like HCTZ did; 1 mg/Kg/day dose (GKW-l group) increased accumulative urine output only at 4-hour time point after water loading; while the highest dose group (20 mg/Kg/day, GKW-h group) did not significantly increase urine output (Fig. 5E,F), some unknown mechanisms maybe involved. GA of 100 mg/Kg/day (GA group) as well as its combination with YHC 0.5 mg/Kg/day (GAYHC-m group) or GKW 5 mg/Kg/day (GAGKW-m group) all had little effect on urine output (Fig. 5G), showing that GA can counteract the diuretic effect of YHC or GKW, which is consistent with the *in-vitro* data. Western blot analysis shows that YHC-h group and GKW-m group both significantly reduced renal total AQP 2 levels (*P* < 0.05), while GA and GAYHC-m group increased AQP 2 levels. When compared to YHC-m or GKW-m group, GAYHC-m and GAGKW-m groups both increased AQP 2 levels (Fig. 5H,I). The AQP 2 expression levels of each group are relatively consistent with the diuretic effect. These data suggest that YHC and GKW are the main compounds in YH for its diuresis activity, and GA (GRA) in GC is responsible for its counteraction on YH’s diuresis effect.

### YHC, GKW and GRA could bind to MEK1/FGFR1 protein in ERK-MAPK pathway

**Figure 6:**
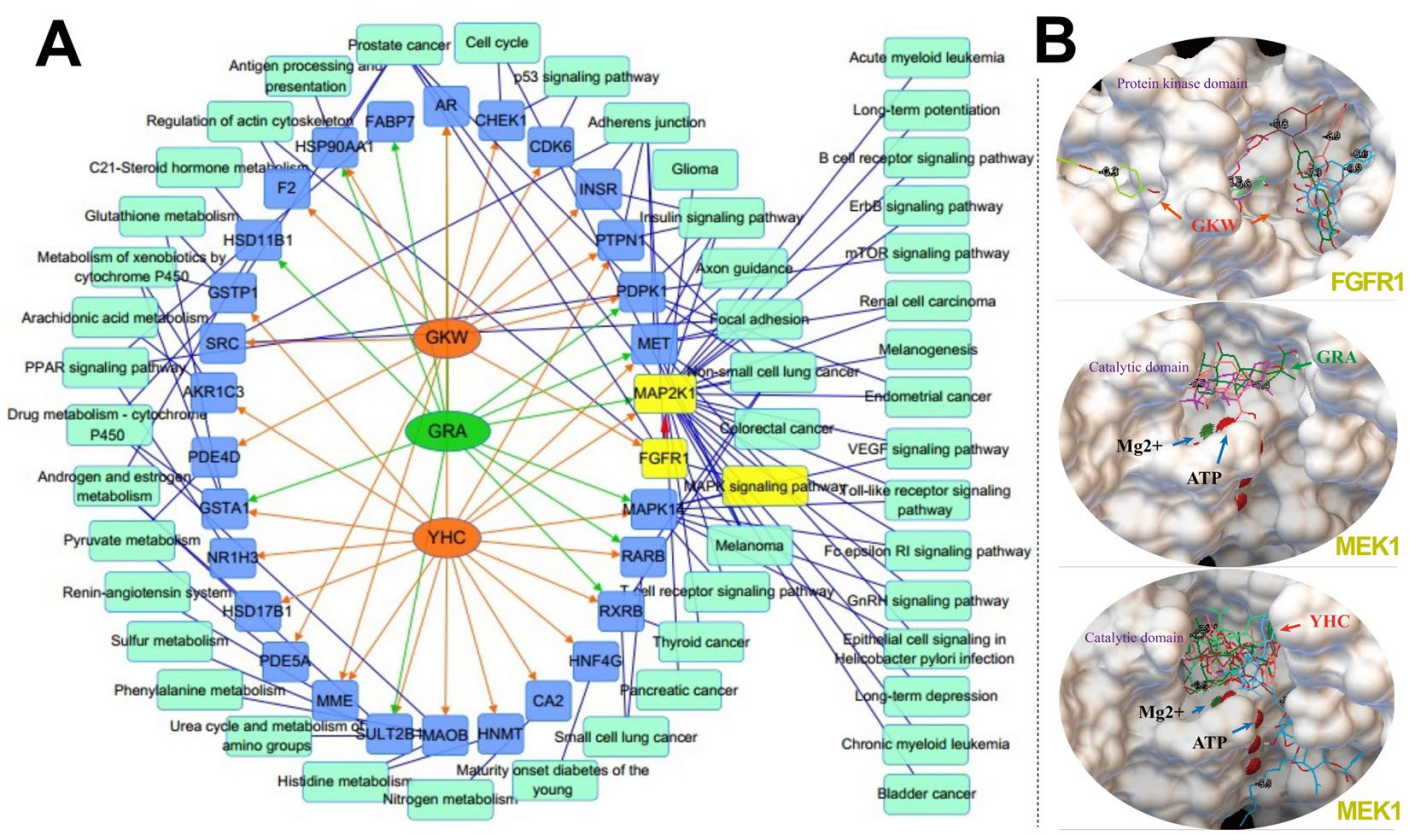
YHC, GKW and GRA may all target on ERK-MAPK pathway. (**A**) Network of targets and pathways (including diseases) interact with YHC, GKW or GRA. Network pharmacology study identified 30 potential targets and 49 potential pathways for these compounds. The targets and pathways linked with GRA were mostly linked with GKW or YHC, which indicate tight interactions between YH and GC. Among the shared targets and pathways, MAP2K1 (equal to MEK1) and FGFR1 are the upstream factors of ERK-MAPK pathway (yellow nodes). This pathway mediates renal AQP 2 expression. Nodes of targets are blue, nodes of pathways are light green, edges linking molecules and targets are orange (YHC and GKW) or green (GRA), edges linking targets and pathways are blue. (**B**) Molecule docking images showing how YHC, GKW and GRA bind to the active domain of their targets, in each case at least 3 to 8 confirmations of different binding mode composed the interactions between ligands and targets (confirmations of the same ligand were merged together). The docking study confirmed the ERK-MAPK pathway, which may be potentially influenced by YHC, GKW and GRA. The active domains were confirmed by GeneCards (http://www.genecards.org/), Mg2+ and adenosine triphosphate (ATP) were coenzymes of MEK1.

We then further study the binding targets or involved pathways of these active compounds, to obtain an overview of how these compounds exert activities or further interact with each other. In order to identify potential target candidates for these compounds, network pharmacology method was used. PharmMapper is an integrated pharmacophore matching platform with over 7,000 receptor-based pharmacophore models extracted from mainstream databases^30^. We used this tool to identify the potential target candidates of YHC, GKW and GRA in these models. Then the targets were submitted to calculate the most connected pathways. Molecular Annotation System is a data-mining platform to extract or analyze biological molecules relationships from public knowledgebase, we employed this tool to construct target-pathway relationships^31^. Results were shown in Fig. 6A, totally 30 targets show up, in which 18 targets were related with YHC, 13 related with GKW and 11 related with GRA. Among the 11 targets for GRA, 9 of them are also targets for YHC or GKW. 49 pathways (including diseases) are considerably picked out, and 23 pathways are linked with GRA. Among the 23 pathways, only 1 of them is not linked with YHC or GKW. These data together indicate the widespread interactions between GC and YH.

The target MAP2K1 (also known as MEK1) and FGFR1 are upstream factors in ERK-MAPK signaling pathway, and it is all connected with YHC, GKW and GRA. This pathway drew our attention since it is related to AQP 2 expression. As reported, AQP 2 expression in collecting duct is dominated by several pathways, the total targets include more than 15 signaling molecules (Supplementary Fig. S8), in which, the ERK-MAPK pathway is one of the most important pathway.

Subsequent molecule docking study again confirmed the interactions between these compounds and MAP2K1/FGFR1 (Fig. 6B). In each case, at least 3 binding mode simultaneously composed the interaction. The docking energy table of each mode is showed in Supplementary Table S1. These data showed that GRA may interact with YHC and GKW through ERK-MAPK signaling pathway and further lead to different AQP 2 expression levels.

### YHC, GKW and GRA could target on ERK-MAPK signaling and regulate p-CREB level

**Figure 7:**
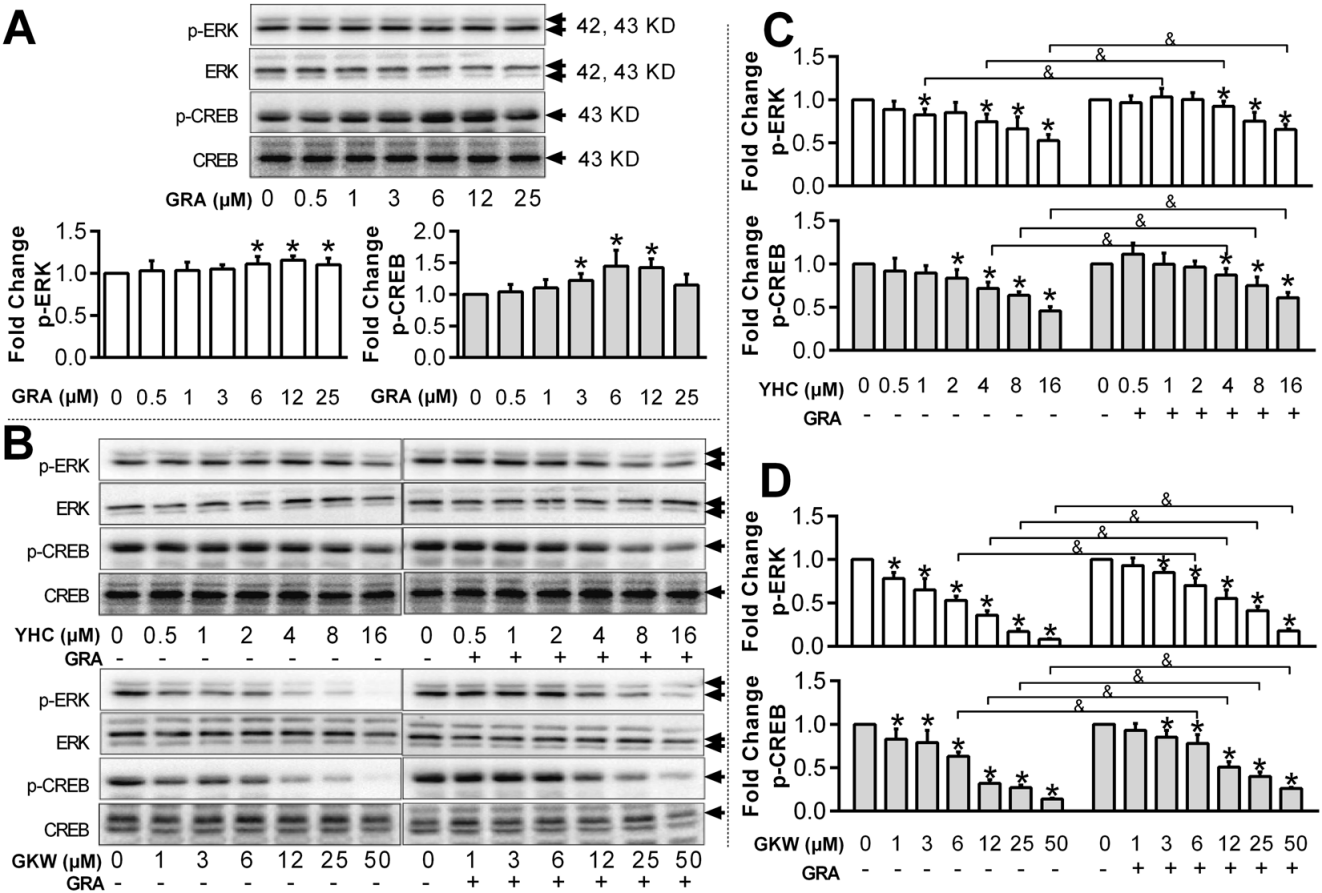
YHC, GKW dose-dependently decrease p-ERK and downstream p-CREB protein expression level, while GRA increase their protein expression level. The mIMCD3 cells were planted in 6 well plate and cultured in RPMI1640 medium supplied with 10% fetal bovine serum. All doses were given during cell logarithmic phase for 24 hours. (**A**) Blotting image of p-ERK, ERK, p-CREB and CREB expressed by mIMCD3 cells treated by GRA. Bar chart of fold changes are given, and concentrations are labeled behind. The blots was cropped properly and the full length blots are presented in Supplementary Fig. S9. (**B**) Blotting image of p-ERK, ERK, p-CREB and CREB expressed by mIMCD3 cells treated by YHC, GKW singly or in combination with GRA, for 24 hours. When GRA was combined, the working concentration was 12 μM. The blots was cropped properly and the full length blots are presented in Supplementary Fig. S9. (**C** to **D**) Bar chart for p-ERK and p-ERK expression with different treatment. *P < 0.05 when compared with control group (none drug-treated), ^&^ *P* < 0.05 compared between groups with or without GRA treatment (n = 3). Total ERK and CREB level were used as internal reference respectively for calculating p-ERK and p-CREB expression. The involvement of ERK-MAPK pathway in AQP 2 expression are showed in Supplementary Fig. S8.

Based on the above data, and according to pathways mediating AQP 2 expression (Supplementary Fig. S8), we hypothesized that YHC, GKW and GRA could target on ERK-MAPK signaling and lead to changes of p-CREB level, the upstream factor of AQP 2 transcription. This hypothesis was further verified by western blotting. In mIMCD3 cells, YHC and GKW down-regulated p-ERK1/2 and p-CREB levels dose-dependently (Fig. 7B–D), while GRA slightly up-regulated p-ERK1/2 and p-CREB (Fig. 7A). As expected, GRA attenuated the effect of YHC or GKW on p-ERK1/2 and p-CREB expression. Figure shows the similar trend between p-ERK1/2 and p-CREB level, suggest tight correlations between p-ERK and p-CREB.

**Figure 8:**
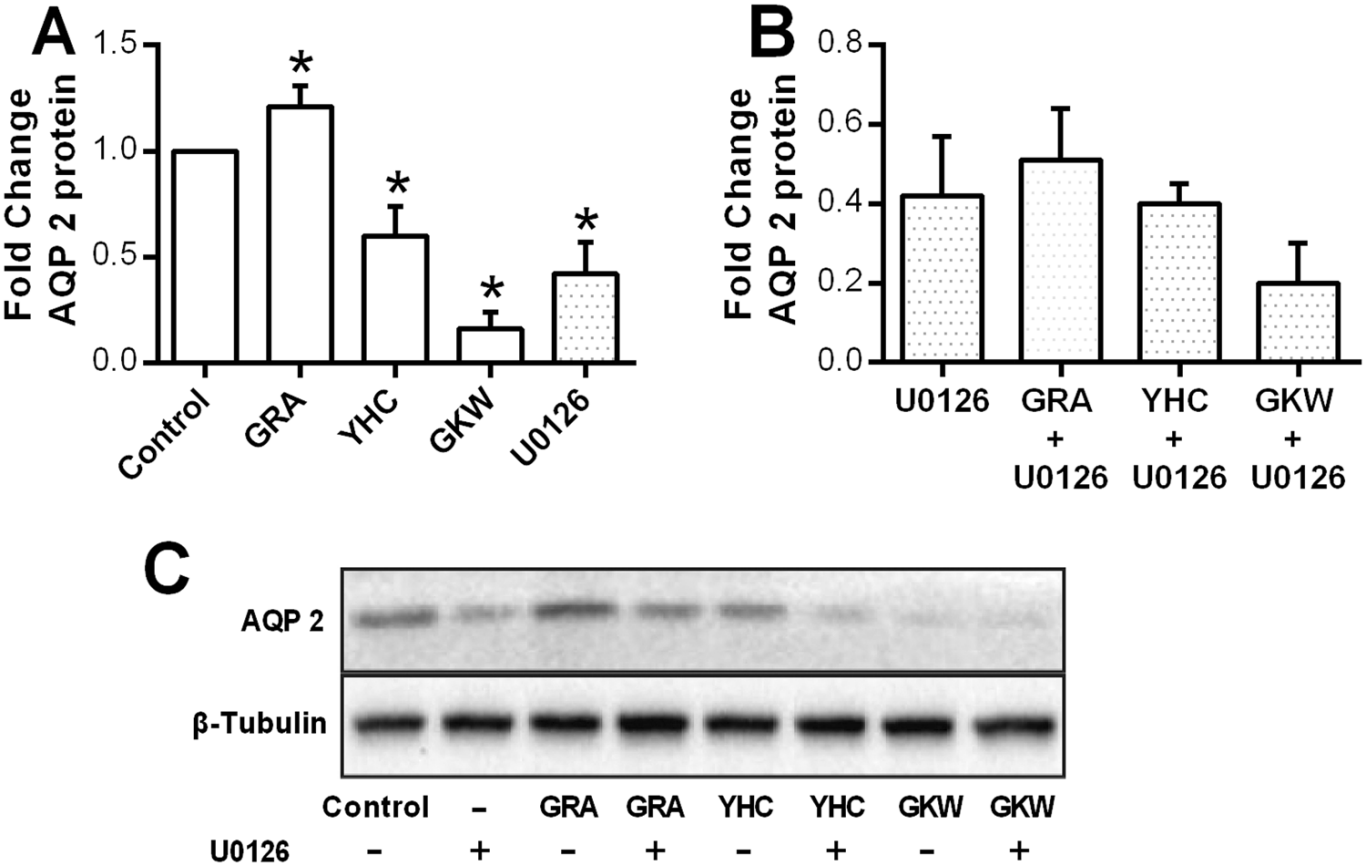
Involvement of ERK-MAPK pathway in AQP 2 regulation by YHC, GKW and GRA was verified by ERK1/2 specific inhibitor. The mIMCD3 cells were planted in 6 well plate and cultured in RPMI1640 medium supplied with 10% fetal bovine serum. All doses were given during cell logarithmic phase for 24 hours. When the ERK1/2 specific inhibitor U0126 was used, cells were pretreated with U0126 for 1 hour and then for another 24 hours with or without YHC, GKW or GRA. YHC, GKW and GRA all changed AQP 2 expression in mIMCD3 cells, however, when the cells were pretreated with U0126, AQP 2 level was suppressed and these compounds’ activity became not significant compared to U0126 single group. (**A**,**B**) Bar chart and (**C**) blotting image of AQP 2 expression of mIMCD3 cells treated with 12 μM GRA, 8 μM YHC and 25 μM GKW for 24 hours, with or without pretreatment with 10 μM U0126. The blots was cropped properly and the full length blots are presented in Supplementary Fig. 10. *P < 0.05 compared with control group (n = 3).

To verify the involvement of ERK-MAPK signaling pathway, specific ERK1/2 inhibitor, U0126 was used. Without U0126, GRA (12 μM) increased AQP 2 expression, while YHC (8 μM) and GKW (25 μM) both decreased AQP 2 expression. When ERK-MAPK signaling pathway was inhibited by U0126 (10 mΜ), AQP 2 protein expresion level also decreased, which proved the important role of ERK-MAPK signaling in AQP 2 regulation (Fig. 8A,C). However, when the cells were pretreated with U0126, these compounds all lost their effects on AQP 2 expression compared with U0126 single treatment (Fig. 8B,C). These data confirmed ERK1/2-CREB-AQP 2 signaling cascade for these compounds.

## Discussion

With concerns about TCM safety and efficiency, the EIM researches have been widely conducted these years, aiming to uncover the mechanisms behind those incompatible herbal pairs, and providing clues for deeper understanding of TCM theories^32–34^. These researches have reminded medical scientists and clinical doctors to re-assess potential problems induced by herbal-herbal or herbal-drug interactions.

In this study, we focused on the diuretic effect of YH when administered with or without GC in diuretic mice model, where GC counteract YH’s diuretic effect. YH increased urine excretion without electrolyte disturbance such as K^+^ loss, which has been observed as side effect of most clinical diuretics including loop diuretics, thiazides and potassium-sparing diuretics. Among the crucial factors influencing urine excretion, renal Aquaporin (AQP) 2 protein was proved to be the key target in YH’s diuretic activity and GC’s counteraction effect. Among the multi-components in YH and GC, YHC, GKW and GRA can be detected in blood and can up or down-regulate AQP 2 expression in mIMCD3 cells. In water loading mice model, YHC and GKW can also promote urine excretion and down-regulate renal AQP 2, while GA can up-regulate AQP 2 and abolish the diuresis effect of YHC or GKW. Furthermore, network pharmacology strategy revealed that all three components can interact with proteins in ERK-MAPK pathway, which is involved in AQP 2 regulation. Western blot analysis further verified that p-ERK1/2 and its downstream p-CREB levels can be regulated by these compounds and further lead to changes in AQP 2 expression. In conclusion, at clinical related doses, GC could counteract YH’s diuretic effect through regulating renal AQP 2 protein expression. GRA (GA) in GC and YHC, GKW in YH are responsible for the AQP2 regulation effect, and ERK-MAPK signaling pathway is also involved.

In clinical practice, doctors usually use diuretic medicines such as spironolactone, HCTZ and furosemide in treating edema, ascites, and hydrothorax. These diuretics target on urine ion reabsorption and may cause electrolyte disturbances such as K^+^ loss or hyperkalemia. Since these diseases are usually complicated with electrolyte disturbances, diuretics like furosemide may further worsen the electrolyte disturbances and have big limitations^35^. Recently, tolvaptan, a novel diuretic targeting AQP 2, has shown superior efficacy in treating ascites. However, it’s also related to hyponatremia and hepatotoxicity^36, 37^. Therefore, the development of diuretics with high safety is urgently needed. In this study, our data shows that YH could exert diuretic effect through modulating AQP 2, but did not influence electrolyte reabsorption and therefore may not lead to electrolyte disturbances, which suggest YH shows advantage to the currently used diuretic drugs and may be developed as a valuable and promising herbal diuretic medicine.

Diterpene esters and flavones are two main type of active compounds in YH. GKW is one of the most abundant flavones in YH, with a content of up to 0.12% in crude drug^38^, while diterpenes like YHC are relatively rare and its hepatotoxicity has been known. This study reveals that GKW and YHC both contribute to YH’s diuretic activity, and GKW may be a safer and more critical active compounds. With high safety and high content, GKW shows a light prospect in diuretic drug development. The renal AQPs have been studied in the diuresis mechanism of herbal diuretics like Fuling and Zhuling^39, 40^. This study shows that AQPs may be a key target for the development of diuresis-related herbs. GA was also reported to up-regulate renal AQP 2 *in vivo*, and the effect was related to the mineralocorticoid receptor^19^, an ion reabsorption regulator. So it can be supposed that GRA could also regulate AQP 2 by osmotic pressure (hypertonicity) *in vivo*.

Our research group have been focused on the study of YH and GC, seeking probable disadvantages caused by their combination^41, 42^. Previously, we found that GA could accelerate the dissolving process of weak polar compounds from herbal pieces when YH and GC were boiled together, the acceleration would probably change the efficacy of YH^43^. In this study, YH and GC were extracted separately before combination, avoiding any interaction happened in boiling procedure. It is also noted that YH was extracted by ethanol, because YH can be taken in powder form in TCM, such as in the famous “Shizao decoction”. GC was extracted by water, in consistent with its application form. What’s more, the doses in the experiments were all designed according to the Chinese Pharmacopeia, making the study consistent with actual situations.

The herbal-herbal interactions exist widely in TCM formulas, involving multi-compounds and multi-targets as well as complicated application situation. This study only focused on the traditional curative effect of YH, the diuresis effect, to study the disturbance of GC on YH, and the compounds studied in these herbs were also limited to relatively high content compounds. The related targets of these compounds were also screened and simplified by *in-silico* strategies. The simplification makes the study of high efficiency but many physiological evidences may be ignored, so lots of work still need to be done for other compounds and for different application situations, such as in tumor treatment, which have been implied by the network pharmacology data. What’s more, advanced research method can also be applied to discover deeper relationships between YH and GC based on the results of this study.

The network pharmacology method has been increasingly engaged in TCM researches, due to its focuses on the multi-component and multi-target features of TCM^44, 45^. In this study, involving compounds was complicated, and pathways were numerous. However, this method largely accelerated the screening of compounds and pathways, proves itself suitable for herbal-herbal or herbal-drug interactions research. Data showed more than 30 targets and more than 49 pathways probably involved in YH-GC interaction. Among the pathways, mainly two aspects stand out- several tumors and metabolism pathways. As for metabolism, GC’s influence on cytochrome P450 enzymes (CYPs) have drawn attentions from scientists^46^. And for tumors, according to Fig. 6A, we can rise a new hypothesis that GC may strengthen or weaken YH in tumor therapies, since chemicals like YHC have been found promising in oncotherapies. More research work has been planned in our team. However, this study focused on AQP 2 regulation, and key targets leading to ERK-MAPK pathway were MEK1 and FGFR1. According to literatures, FGFR1 is important in kidney development^47, 48^ and can be found in collecting duct cells of adult kidneys^49^. FGFR1 and MEK1 are both part of ERK-MAPK pathway in collecting duct cells, this information is critical for us to link YHC, GKW or GRA with AQP 2 through ERK-MAPK pathway. Although YHC, GKW and GRA all target ERK-MAPK pathway, their regulations on p-ERK1/2 levels are entirely different. We did not measure all factor levels in this pathway, and the detailed mechanism still remains unknown, but literatures have indicate that GRA could up or down-regulate ERK-MAPK pathway in different situations^50, 51^.

Finally, we show the possibility in which impaired efficacy can be induced by specific combination of herbs. This possibility gives support to the EIM theory of TCM, indicating that when Yuanhua was used to treat diseases caused by retained morbid fluid including edema, hydrothorax, and ascites, Gancao should not be used in combination. The study reminds that more attention should be paid to the safety and efficacy problems induced by improper combination of modern drugs, natural products and herbs. Also, our work gives an example of systemic herbal-herbal interaction research, from whole body to organs, to cells and molecule targets, step by step. Moreover, it’s revealed that Yuanhua and its active component GKW shows a light prospect in diuretic drug development.

## Material and Methods

### Herbal medicine

Herbal pieces of YH and GC were purchased from Anhui BBCA Pharmaceutical Co., Ltd. and the material were authenticated by the corresponding author as Genkwa Flos (flower buds from *Daphne genkwa* Sieb. et Zucc.) and Glycyrrhizae Radix et Rhizoma (originating from *Glycyrrhiza uralensis* Fisch.), and the voucher specimen of YH (No. 110216) and GC (No. 110701) were deposited at the Herbarium in Jiangsu Key Laboratory for High Technology Research of TCM Formulae, Nanjing University of Chinese Medicine. The UPLC-TOF-MS profile of YH was acceptable with several chemicals identified by the method established by our laboratory (Supplementary Fig. S11)^28^. Liquirtin and GA in GC were determined as 0.87% and 3.6% respectively.

YH pieces was boiled with 10 times’ volume of 95% ethanol under reflux for 2 hours, and was extracted for 3 times. The extract was filtered and rotary evaporated to remove residual ethanol, stored under −20 °C until use, and was suspended with NS to needed doses before orally administrated. Similarly, GC was extracted with distilled water under reflux for 2 hours, 3 times, and diluted with NS. YG was achieved through mixing the extracts of YH and GC followed by diluting with NS.

### Experimental animals

Adult male ICR mice weighting between 18 and 22 g were obtained from the Comparative Medicine Center of Yangzhou University (License No: SCXK 2012-0004) and used in the experiments after 7 days of acclimatization. All experimental protocols including the care and handling of mice were approved by the Animal Ethics Committee of Center for Drug Safety Evaluation and Research of Nanjing University of Chinese Medicine (Nanjing, China). The methods were carried out in accordance with the Guiding Principles for the Care and Use of Experimental Animals from the committee in accordance with the internationally accepted standard guidelines for use of animals. The mice were kept in a temperature controlled environment (22 ± 2 °C), 55% ± 5% relative humidity with a 12-h: 12-h light-dark cycle and fed with standard chow.

### Experimental design

ICR mice were divided randomly into 9 groups with equal number (n = 15): The normal group (N), model group (water loading with saline injection, M), HCTZ group, YH high dose group (YH-h), YH low dose group (YH-l), GC high dose group (GC-h), GC low dose group (GC-l), YH high dose plus GC high dose group (YG-h) and YH low dose plus GC low dose group (YG-l). The N and M groups of mice were orally administrated by syringe feeding with NS (10 mL/kg/day, the same volume as all other groups), the other groups were orally administrated with HCTZ (7.5 mg/kg/day), YH high or low dose (0.28 or 0.14 g crude drug/kg/day), GC high or low dose (1.5 or 0.75 g crude drug/kg/day), YG high or low dose. All high doses were equal to the upper limits of the Chinese Pharmacopeia. HCTZ was used as the reference drug to verify the diuretic activity tests. It was suspended in NS prior to administration. In another independent experiment, animals were treated like above, and YHC of 1, 0.5 and 0.1 mg/Kg/day doses (YHC-h, YHC-m, and YHC-l groups respectively) as well as GKW of 20, 5, and 1 mg/Kg/day doses (GKW-h, GKW-m, and GKW-l groups respectively) were examined in the model. HCTZ (7.5 mg/kg/day), GA (100 mg/kg/day) and its combination with YHC-m and GKW-m doses were also examined for comparison.

The administration of drugs was implemented once a day for 3 days. On the fourth day all mice except for N group were weighted and intraperitoneal injected with NS of 15% body weight^17, 52, 53^. Drinking water was revoked and the mice were immediately weighted every hour for 8 hours. Mice were placed individually in a small stainless-steel cage on a glass dish and urine was gathered and combined every 10 minutes. In one experiment, at the time point of 4-hour after water-loading, animals were sacrificed and plasma was collected. High dose groups of YH, GC, YG and the N, M, HCTZ groups, as well as YHC, GKW and GA groups were selected, and animal kidneys were removed and cut into pieces, snap frozen in liquid nitrogen and kept at −80 °C.

### Evaluation of diuretic effect

After water-loading, mice urinated, and their weight loss were measured every hour, DI representing urine output for each mouse was calculated as the percentage of weight loss in total loaded water. The data were expressed as mean with standard deviation for each group. Another parameter, urine osmolality was measured to reflect urine concentrating ability, and additionally, UCF was calculated to indicate the water reabsorption percentage. Based on the fact that plasma Cr is filtered to the primary urine without a concentration change, then the primary urine is reabsorbed without Cr reabsorption, so the UCF can be calculated as urine/plasma Cr concentration ratio^54^. Total amount of secreted electrolytes were also standardized with urine Cr. Given that bodies generate and excrete Cr at a relatively constant speed, the total amount of excreted urine Cr is relatively constant. So the amount of urine electrolytes excretion can be standardized by Cr excretion amount. Data was calculated as electrolytes/Cr concentration ratio^55, 56^. We adopt plasma Cr clearance (Ccr) as estimated GFR^57^, the Ccr was calculated as urine Cr multiplied with urine volume and divided with plasma Cr.

### Measurement of Cr, electrolytes, Osmolality, AVP, and ALD

Urine Cr, plasma Cr and urine Na^+^, K^+^, Cl^−^ concentration were measured with automatic biochemical analyzer (Hitachi 7020P, Japan). Reagent kits for Cl^−^ concentration was based on Mercury Thiocyanate method; the Cr, Na^+^ and K^+^ concentration were based on enzyme methods, and were used according to the manuals. Urine osmolality was measured by Osmometer (Fiske 210, USA).Plasma AVP and ALD were measured with ELISA kits, all the kits were obtained from Sangon Biotech Shanghai Co., Ltd. (China).

### Cell culture and treatment

The mIMCD3 cells (mouse inner medullary collecting duct cells) were purchased from ATCC, cultured with RPMI 1640 medium containing 10% fetal bovine serum, incubated in 37 °C provided with 5% CO_2_ and 95% air. Before treatment, the cells were planted into 6 well plates until adhering to the bottom. Then culture medium was replaced and drug containing medium was added, incubating for 24 hours and cell number was multiplied. After rinsing with phosphate buffered saline for 3 times, cells were kept in −80 °C refrigerator.

### The network pharmacology study and molecule docking validation

Three dimensional confirmation file of YHC, GKW and GRA were downloaded at the site of PubChem Compound (NCBI), with SDF file format, their CID numbers are shown above. These files were imported into PharmMapper Server^30^. (http://59.78.96.61/pharmmapper/index.php), target set was limited in human proteins, and the other parameters were kept as default. The top 30 targets with higher statistical “Fit” number was chosen, further, their Uniprot accession number were submitted to Molecule Annotation System (MAS 3.0, http://bioinfo.capitalbio.com/mas3/) to search possible pathways against KEGG database in human spices^31^. In final results, the top 30 possible pathways with *P* value less than 0.01 was showed by Cytoscape software (version 3.3.0).

Next, molecular docking study was conducted to show how small molecules interact with selected targets. The ligand molecule file (MOL2 file) for YHC, GKW and GRA were downloaded from TCMSP database^58^, and the receptors were downloaded from RSCB PDB database (http://www.rcsb.org/pdb/home/home.do), with ID number of “1s9j” for MEK1 protein and “3c4f” for FGFR1 protein. The receptors were prepared by deleting water and ligand, adding polar Hydrogens and “Kollman” charges with AutoDockTools (version 1.5.6). Docking was completed in AutoDock Vina^59^, parameters were all kept as default according to software manuals.

### Protein isolation and western blotting analysis

For each kidney sample, about 100 mg tissue was extracted with RIPA lysis-buffer. For mIMCD3 cells, 150 μL RIPA was added per well. Protein concentrations were measured by Bradford protein assay, and about 50 μg proteins were loaded on a 12% or 15% of SDS-polyacrylamide gel for separation and then transferred onto polyvinylidene fluoride membrane. Membranes were blocked with 5% bovine serum albumin in a solution [50 mM Tris-HCl, 150 mM NaCl, 0.1% v/v Tween-20, pH 7.6 (TBST)] for 2 hours, then incubated overnight at 4 °C with primary antibodies. After eight 5-min washing with TBST, the blots were incubated with secondary antibody at room temperature for 1 hour. Washing as above, the targeted band was visualized using the enhanced chemi-luminescence system. Polyclonal antibodies to AQP 1 and 3 were obtained from Santa Cruz Biotechnology (USA), polyclonal antibodies to AQP 2 phospho S269 was obtained from GeneTex (USA), polyclonal antibodies to AQP 2 phospho S256 and phospho S261, AQP 4 and AVPV2R, were from Abcam (USA), and polyclonal antibody to AQP 2, ERK1/2, p-ERK1/2, CREB, p-CREB and β-tubulin was purchased from Cell Signaling Technology (USA). Secondary antibody was from Proteintech (USA).

### Statistical analysis and image acquisition

All data is expressed as means ± standard deviation. After confirming the normality of the data, statistical significance of differences was evaluated by one-way analysis of variance (ANOVA) followed by Dunnett’s T3 test (SPSS11.5). *P* < 0.05 was considered as statistically significant, and when *P* < 0.01, the *P* value is claimed in the main text. The blotting bands were visualized and quantifed via densitometry using ChemiDoc XRS+ system and inner software. The bar chart and blotting images are combined with GraphPad Prism 6 software. Schematics are drawn by Adobe Illustrator software.

### Data Availability

The datasets generated during and/or analysed during the current study are available from the corresponding author on reasonable request.

## Electronic supplementary material

Supplementary Information
