# Supplementary material for: How impaired efficacy happened between Gancao and Yuanhua: Compounds, targets and pathways
Source: Sci Rep. 2017 Jun 19;7:3828. doi: 10.1038/s41598-017-03201-1 (PMC5476574; doi:10.1038/s41598-017-03201-1)
Supplement: Supplementary file 1 — Supplementary Information [file 41598_2017_3201_MOESM1_ESM.pdf]

How impaired efficacy happened between Gancao and Yuanhua: Compounds, targets and pathways

Jin-gao Yu [1, 2], Jianming Guo [1, 2*], Kevin, Yue Zhu [1, 2*], Weiwei Tao [1, 2], Yanyan Chen [1, 2], Pei Liu [1, 2], Yongqing Hua [1, 2], Yuping Tang [1, 2], Jin-ao Duan [1, 2*]

[1] Jiangsu Collaborative Innovation Center of Chinese Medicinal Resources Industrialization, and National and Local Collaborative Engineering Center of Chinese Medicinal Resources Industrialization and Formulae Innovative Medicine, Nanjing University of Chinese Medicine, 138 Xianlin Road, Nanjing, 210023, Jiangsu Province, China. [2] Jiangsu Key Laboratory for High Technology Research of TCM Formulae, Nanjing University of Chinese Medicine, Nanjing 210023, China.

[*] Co-corresponding author, e-mail address: dja@njucm.edu.cn (**J. Duan**); njuguo@njucm.edu.cn (J. Guo); nzyzy808@163.com (K.Y. Zhu).

Supplementary Information

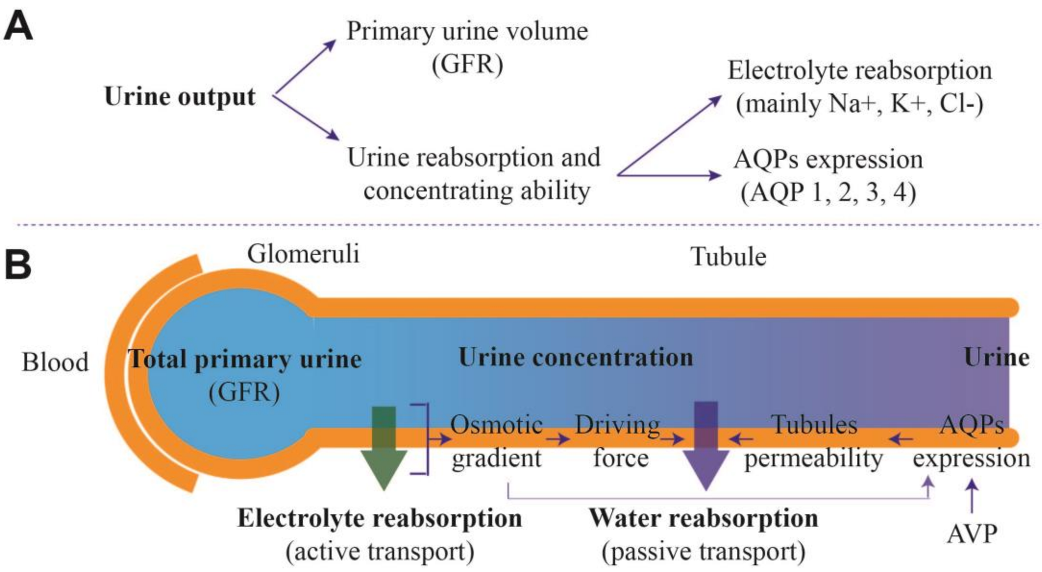

Supplementary Fig. S1. Key factors in urine formation process which has been investigated in this study. (A) Decomposition of factors determining urine output. GFR, urine concentrating ability, urine ion concentration and renal AQPs were studied step by step. (B) Urine formation process. Blood was filtered by glomeruli to generate primary urine, the filtering rate known as GFR reflect the total primary urine volume. Then the water in primary urine will be reabsorbed by kidney tubules, the osmotic pressure decided by electrolyte reabsorption provide driving force for water transport,

while renal AQPs expression control the permeability of tubules. AQPs are regulated by hormones like AVP and by tonicity produced by electrolyte reabsorption [1–3].

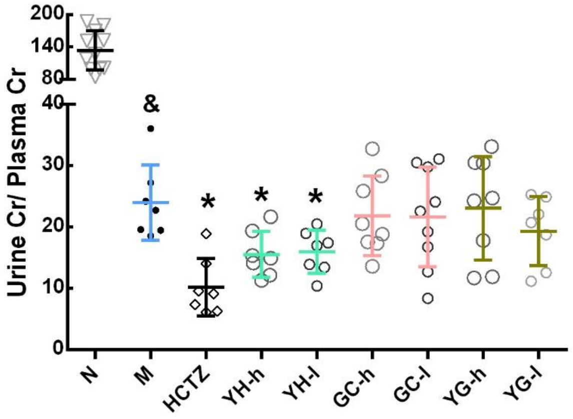

Supplementary Fig. S2. Water reabsorption was reflected by UCF and calculated by urine/ plasma Cr ratio (n= 7-11). Higher UCF means more water was absorbed from primary urine. Water loading itself largely decreased UCF, HCTZ and YH could further lower it, whereas GC and YG had no effect.

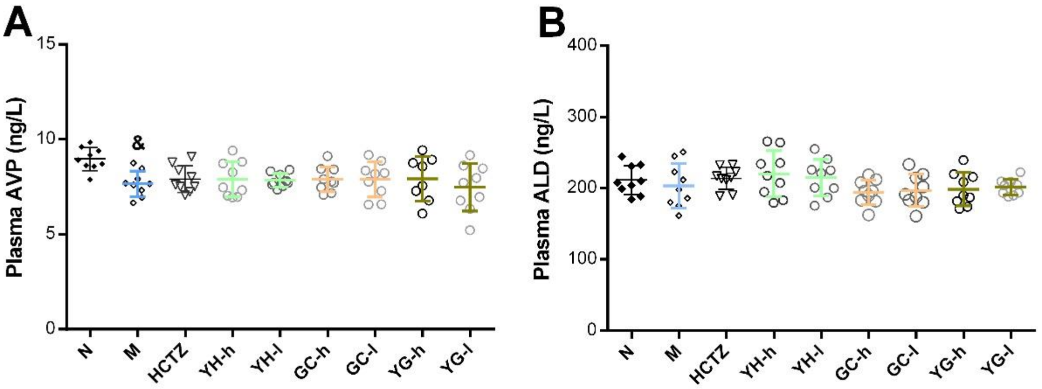

Supplementary Fig. S3. Plasma AVP and ALD of mice 4 hours after water loading. They are the two main hormones in water-electrolyte metabolism. (A) Plasma AVP and (B) plasma ALD concentrations (n= 9-11). & $P < 0.05$ compared to N group. NS loading significantly reduces plasma AVP, responding to water excess. ALD was not affected in all groups.

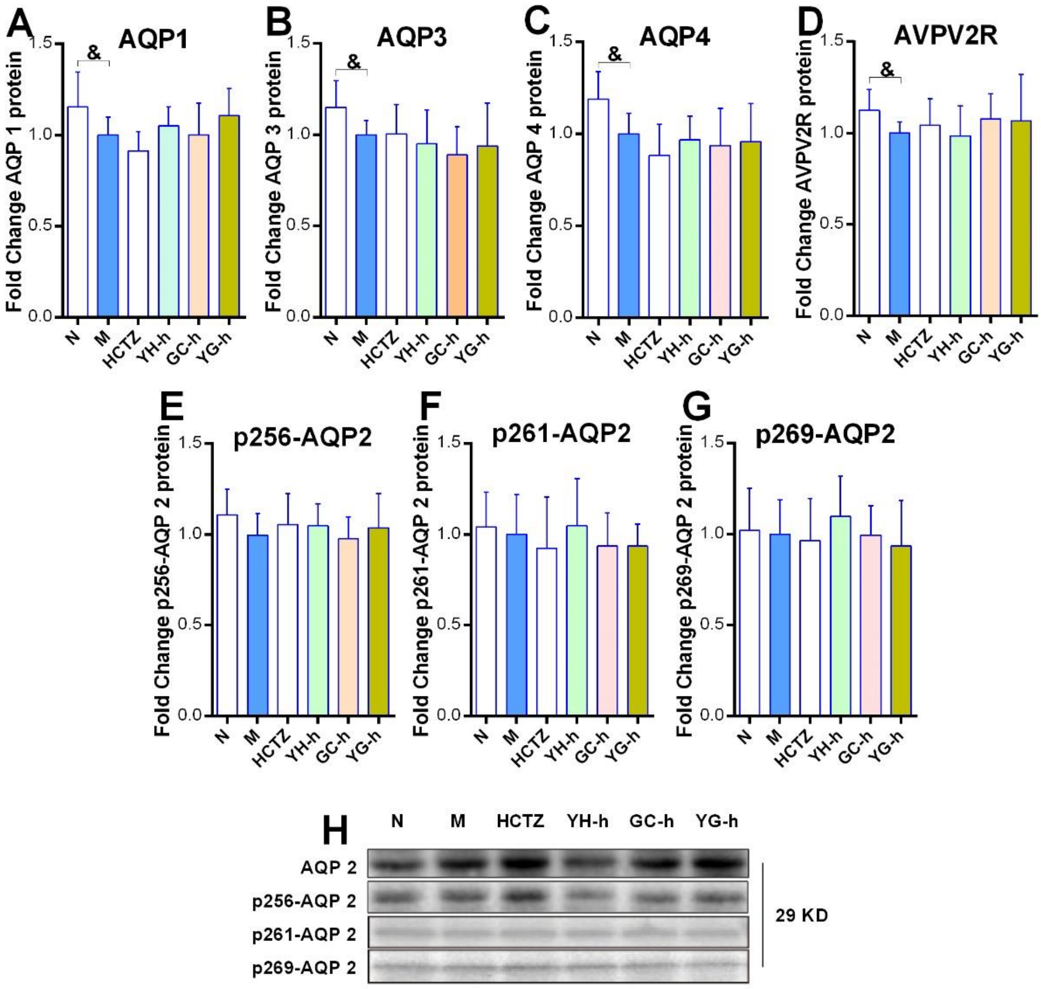

Supplementary Fig. S4. Some main factors involving in water reabsorption in kidney, which were not regulated by YH or GC. (A to G) Bar chart of renal AQP 1, 3, 4, AVPV2R, p256-AQP 2, p261-AQP 2, and p269-AQP 2 expression of animal kidneys. Average number of M group is set as 1.0. NS loading significantly down-regulate AQP 1, 3 and 4 and AVPV2R. & $P < 0.05$ (n= 6). When calculating p-AQP 2 expression, AQP 2 was used as internal reference. (H) Blotting images of phosphorylated AQP 2 proteins, including p256-AQP 2, p261-AQP 2 and p269-AQP 2 in different treatment groups. Total AQP 2 was used as internal reference. One representative sample of each group is showed.

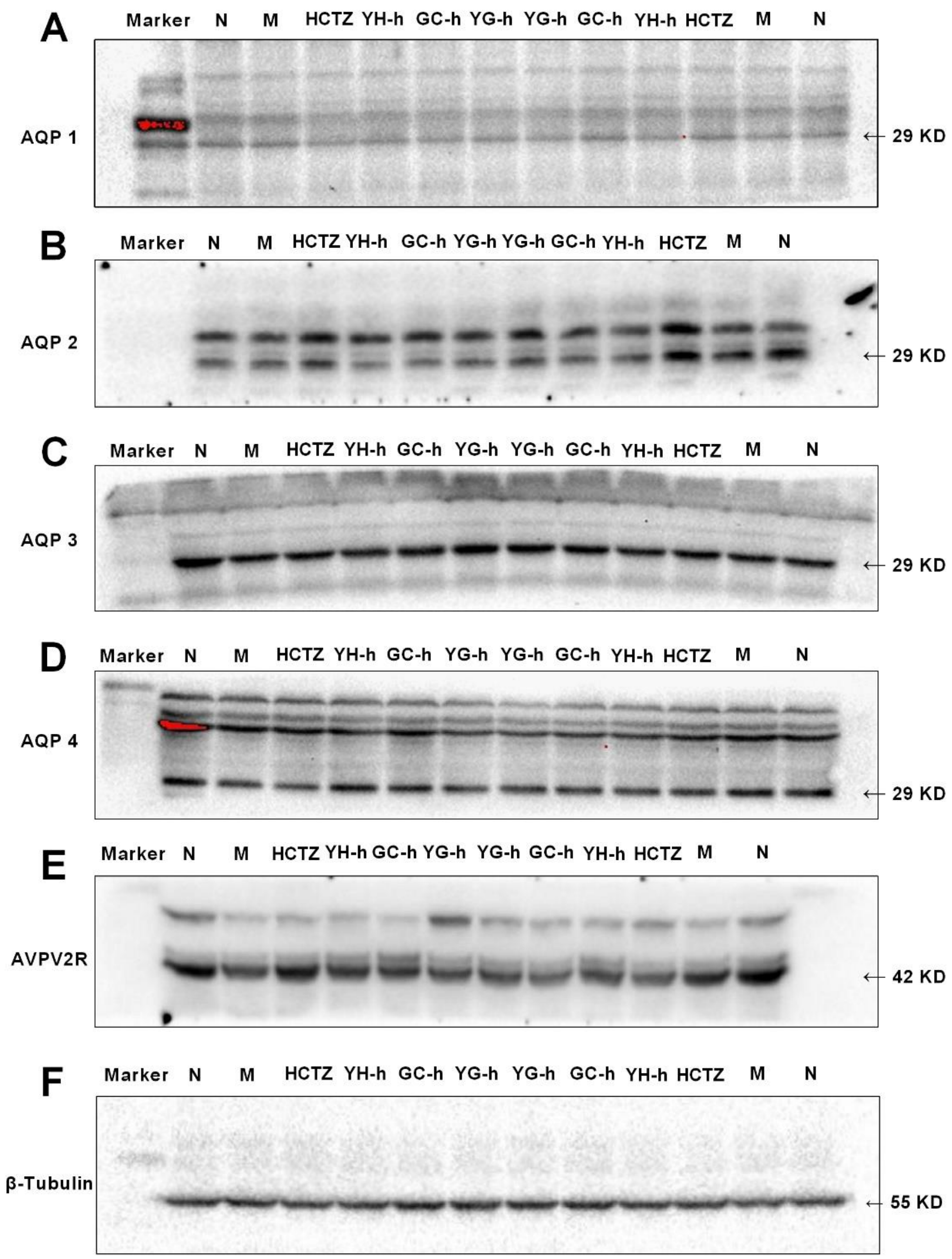

Supplementary Fig. S5. Uncropped blots of diuresis related proteins in animal kidney. (A to F) AQP 1, AQP 2, AQP 3, AQP 4, AVPV2R and β-Tubulin protein expressions in each treatment groups. According to diuresis evaluation results, high doses of YH, GC and YG were selected for western blotting study, and two representative sample of each group are showed. Each protein in the blot is recognized by its molecular weight. For AQPs, we studied its un-glycosylated form of 29 KD band since there are too many glycosylated bands in each lane according to the antibody manual. The group names are given above the blots.

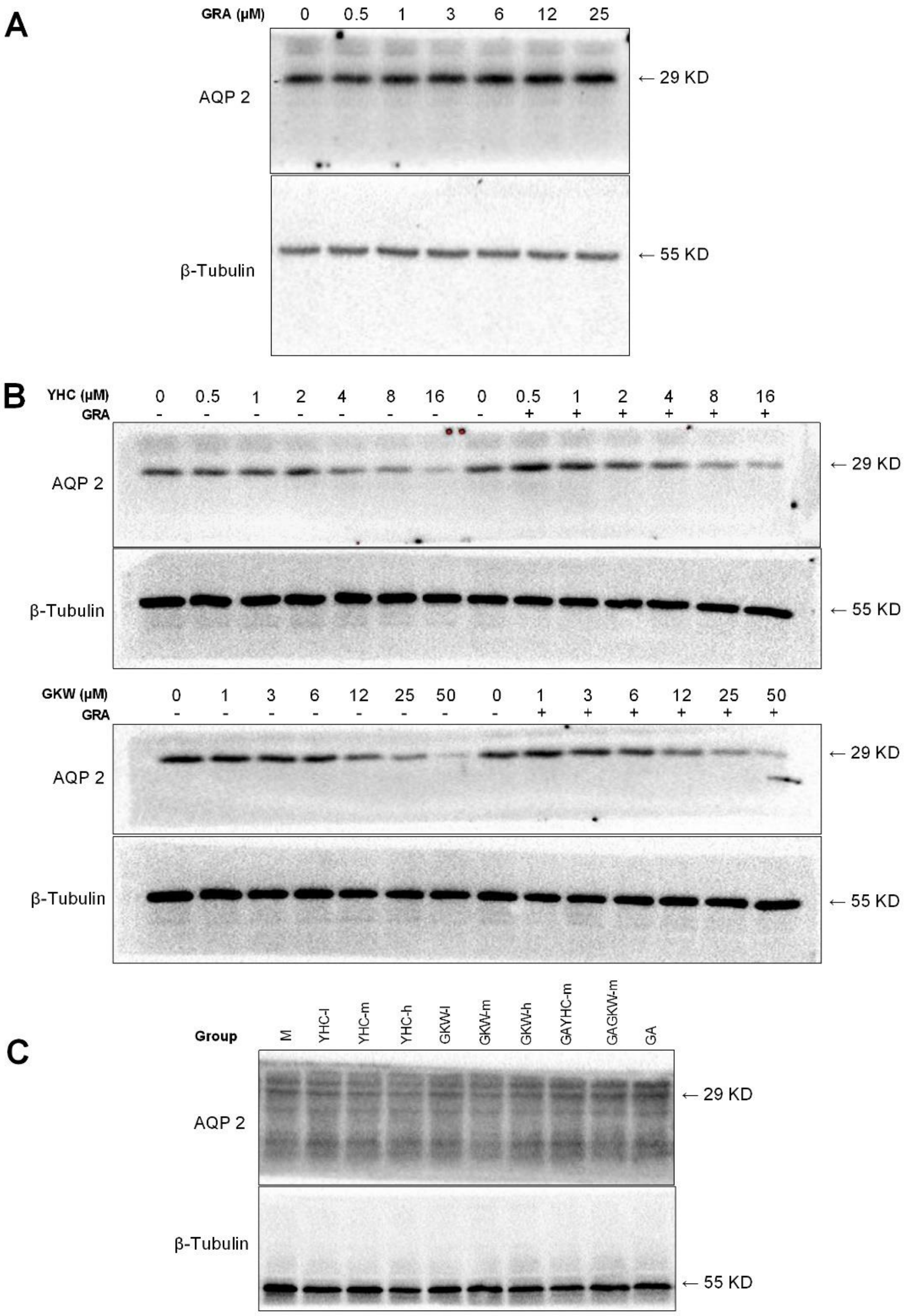

Supplementary Fig. S6. Uncropped blots of AQP 2 and β-Tubulin in-vitro and in-vitro. (A and B) AQP 2 expressions in mIMCD-3 cells treated by GRA, YHC, and GKW. (C) AQP 2 expressions in animal kidney treated by YHC, GKW, GA and their combinations for 4 days. The 29 KD bands were recognized by molecular weight according to protein markers. The doses or group names are given above each blot.

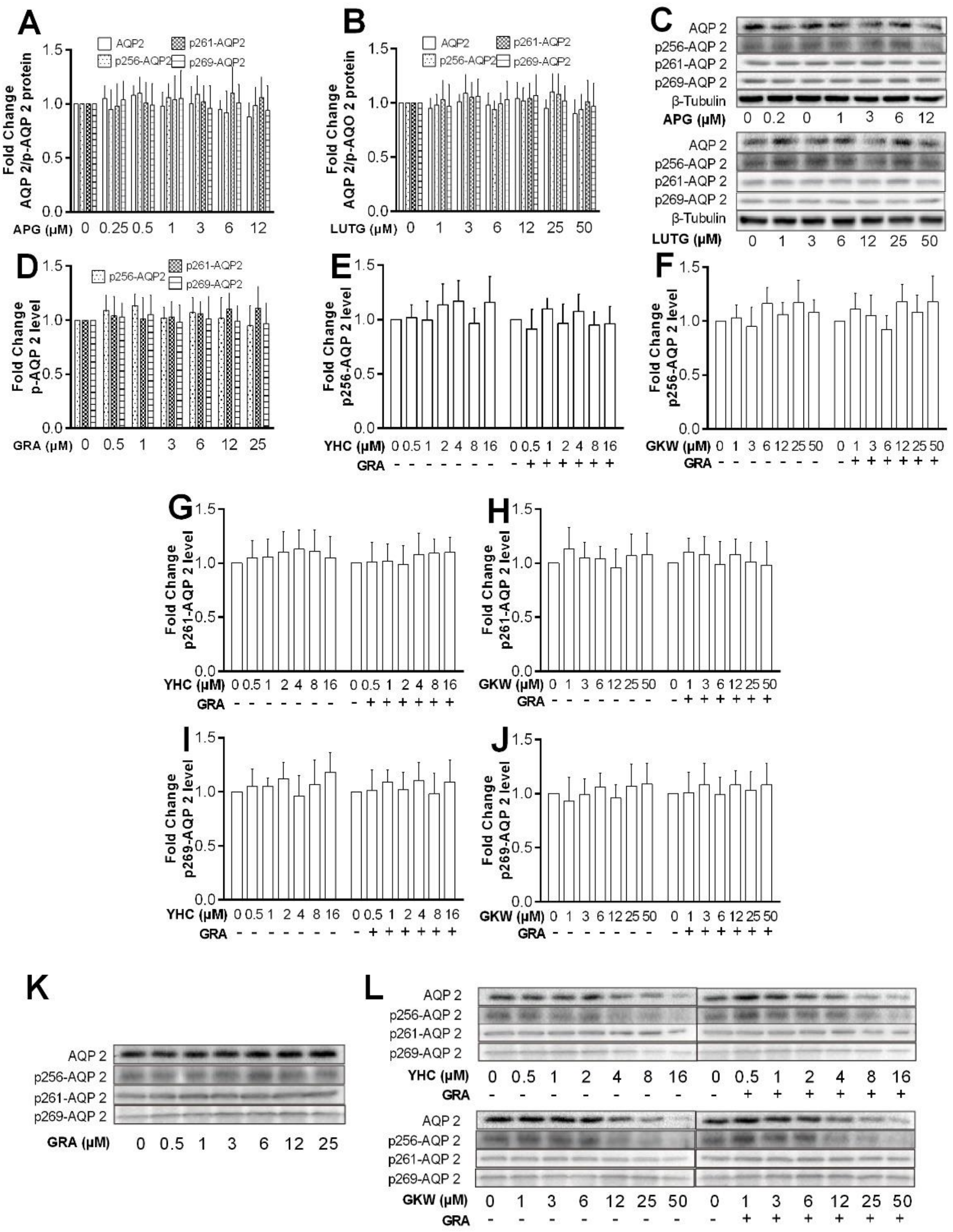

Supplementary Fig. S7. APG or LUTG may not be active compounds of YH in AQP 2 regulation and AQP 2 phosphorylation level cannot be changed by GRA, YHC or GKW. (A and B) Bar chart for AQP 2 and p-AQP 2 expressions of mIMCD3 cells treated by APG or GKW (n= 3). (C) Blotting image of AQP 2 and p-AQP 2 of mIMCD3 cells treated by APG or GKW. Concentrations are labeled behind. (D to J) Bar chart for p-AQP 2 expressions (including p256-AQP 2, p261-AQP 2, p269-AQP 2) of mIMCD3 cells treated by YHC, GKW and GRA singly or in combination (n= 3).When GRA was combined, the working concentration was 12 μM. When calculating p-AQP 2 expressions, AQP 2 was used as internal reference. The phosphorylation levels of AQP 2 are not involved in the regulation effect of YHC, GKW and GRA. (K and L) Blotting

images of phosphorylated AQP 2 proteins, including p256-AQP 2, p261-AQP 2 and p269-AQP 2 in mIMCD-3 cells treated by GRA, YHC and GKW. Total AQP 2 was used as internal reference. The doses are marked below the charts or blots.

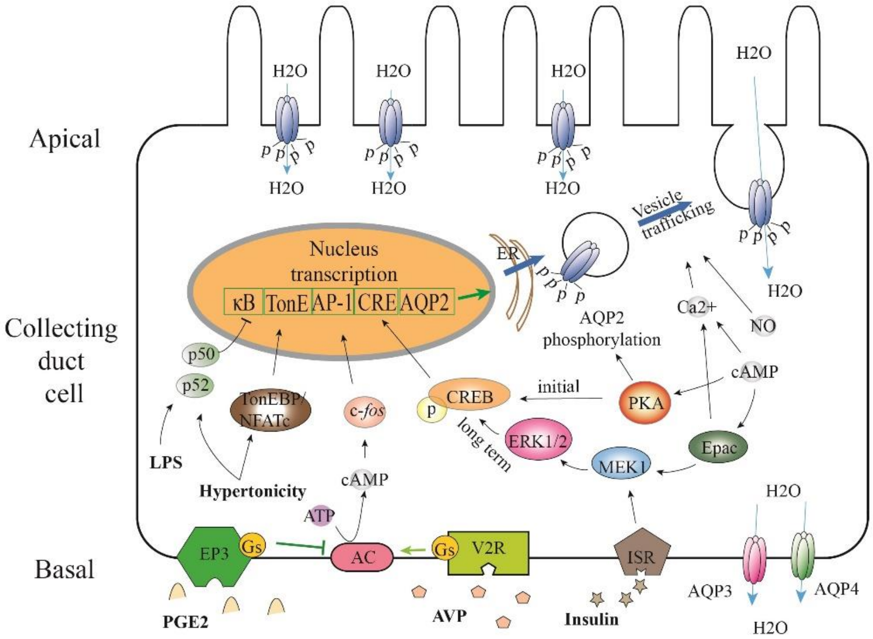

Supplementary Fig. S8. Signaling pathways involving in renal AQP 2 regulation. AQP 2 expression and traveling in renal collecting duct cells are regulated by AVP, insulin, hypertonicity and Prostaglandin E2 (PGE2), among which AVP is the most dominant. cAMP-PKA-CREB signaling mediates shot-term AQP 2 expression and phosphorylation and exocytosis to apical membrane [4], while cAMP-Epac-ERK1/2-CREB signaling mediates long-term AQP 2 expression [5,6]. PGE2and its EP3 receptor are believed to inhibit adenylate cyclase (AC) [7], and insulin up-regulate AQP 2 through ERK-MAPK pathway after binding to insulin receptor (ISR) [8]. Hypertonicity and lipopolysaccharide (LPS) also regulate AQP 2 by NF-κB or by Tonicity-responsive enhancer binding protein (TonEBP/NFATs) [9,10]. Nitric oxide (NO), calcium ions (Ca^{2+}) and other factors may also take part in AQP 2 regulation [4]. V2R or AVPV2R, arginine vasopressin receptor V2; ER, endoplasmic reticulum; -p, phosphorylation.

Supplementary Table S1. Docking energy of top 9 confirmations for YHC, GKW or GRA, when they are binding to MEK1 or FGFR1.

	YHC			GKW			GRA		
Binding Mode	Affinity kcal/mol	RMSD distance from best mode		Affinity kcal/mol	RMSD distance from best mode		Affinity kcal/mol	RMSD distance from best mode	
		Lower bond	Upper bond		Lower bond	Upper bond		Lower bond	Upper bond
1	-7.5*	0	0	-7.4	0	0	-8.3*	0	0
2	-7.1*	1.518	2.226	-7.3*	11.77	13.88	-7.7*	2.643	4.285

3	-7.1*	3.397	7.098	-6.9*	14.53	17.15	-7.6	27.16	30.99
4	-6.8*	2.062	3.449	-6.9*	13.79	15.96	-7.5	27.13	31.50
5	-6.8	29.49	33.35	-6.8*	17.43	19.81	-7.4*	1.700	8.512
6	-6.8*	10.78	14.43	-6.7*	17.41	18.83	-7.4	25.35	28.56
7	-6.7*	1.925	4.499	-6.6*	14.91	17.44	-7.3	25.12	27.53
8	-6.7	27.34	30.88	-6.6*	16.15	18.81	-7.3	25.39	28.13
9	-6.7	27.01	30.69	-6.3*	22.78	26.06	-7.2	24.51	26.97

* Confirmations showed in Fig. 6B. RMSD distances represent the differences between specific binding mode and the top 1 binding mode [11].

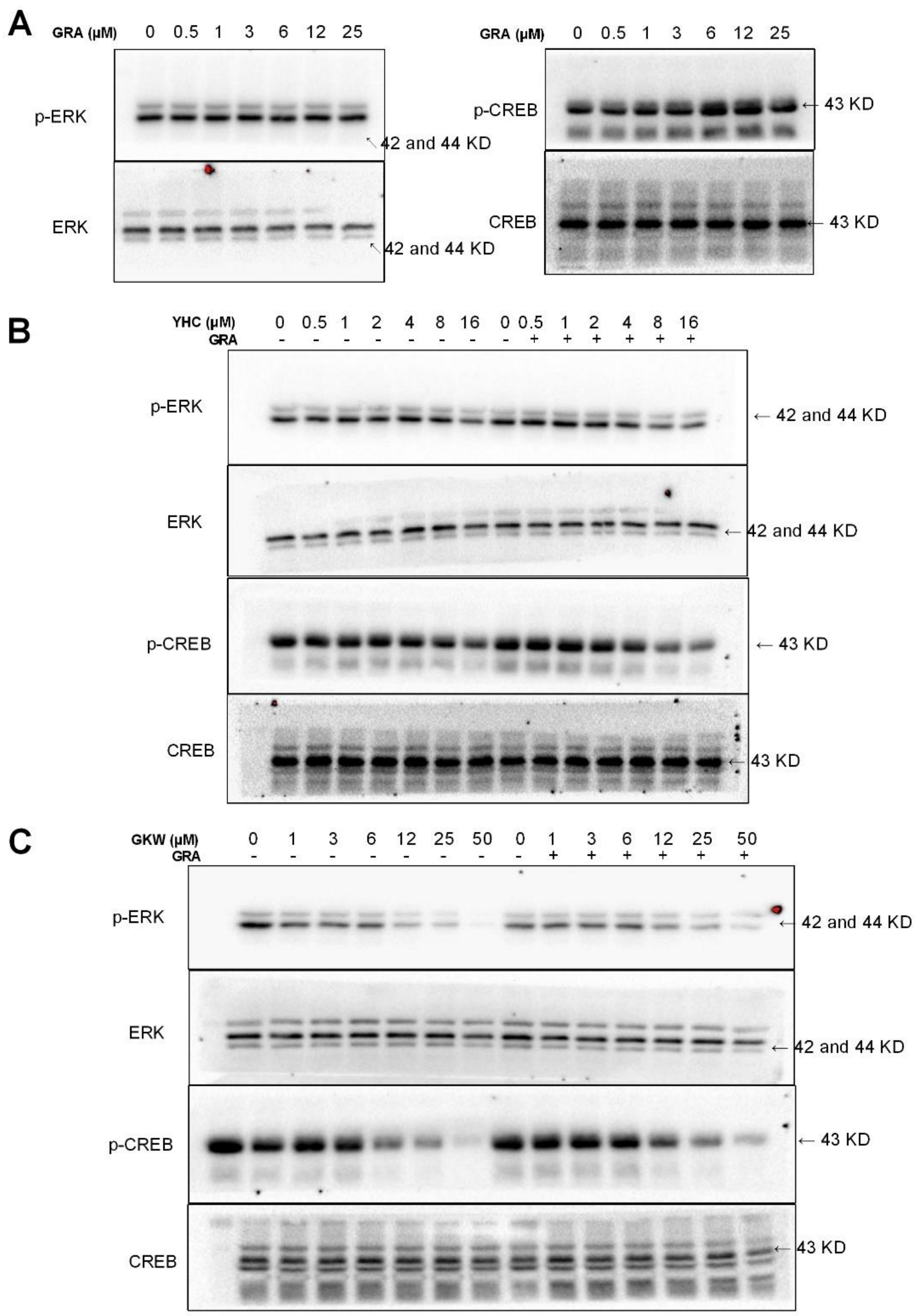

Supplementary Fig. S9. Uncropped blots of p-ERK and p-CREB expressions in-vitro. (A to C)

p-ERK and p-CREB expressions in mIMCD-3 cells treated by GRA, YHC, and GKW. The total ERK and CREB are used as internal references. The 42 and 44 KD bands were recognized as ERK 1 and ERK 2. The 43 KD bands are CREB, it can be distinguished from the interfering bands by its strength and relative position, according to the antibody manual. The doses are given above each blot.

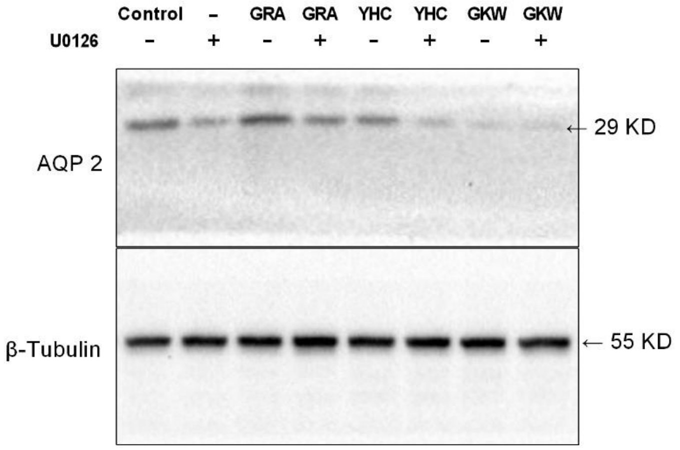

Supplementary Fig. S10. Uncropped blots of AQP 2 in mIMCD-3 cells. The cells were pretreated with U0126 for 1 hour and then for another 24 hours with or without YHC, GKW or GRA. The group names are marked above the blot.

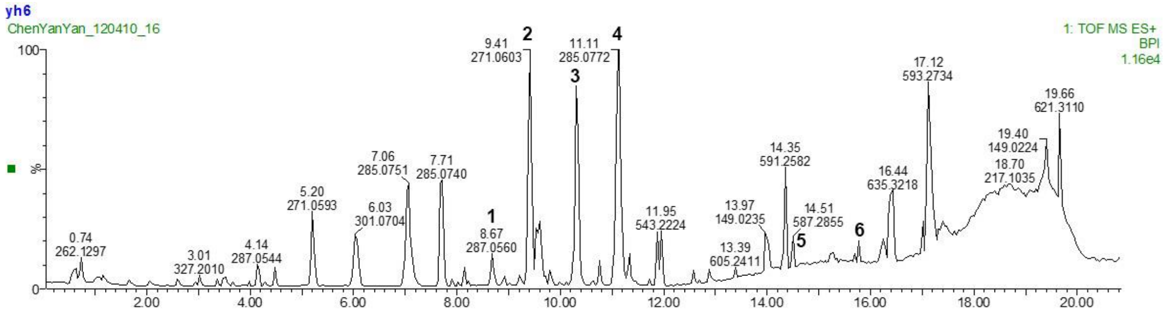

Supplementary Fig. S11. Chemical profile of YH used in this study. UPLC-TOF-MS profile of YH (95% ethanol extraction) was obtained in positive ion mode, the Thermo Syncronic C18 column (100 mm × 2.1 mm, 1.7 μm) was used and the mobile phase was composed of A (0.1% formic acid) and B (acetonitrile) eluting with the gradient: 0 - 3 min 5% B, 3 - 5 min 5 - 15% B, 5 - 9 min 15% B, 9 - 16 min 15 - 70% B, 16 - 20 min 70 - 95% B, 21 - 22 min 5% B, flow rate was set at 0.4 mL/min. The chemicals identified were Luteolin (1), Apigenin (2), 3'-Hydroxygenkwanin (3), Genkwanin (4), Yuanhuadine (5), Yuanhuacine (6).

References

1. Sands, J. M. Urinary Concentration and Dilution in the Aging Kidney. *Semin. Nephrol.* **29,** 579–586 (2009).

2. Jung, H. J. & Kwon, T.-H. Molecular mechanisms regulating aquaporin-2 in kidney collecting duct. *Am. J. Physiol. Renal Physiol.* ajprenal.00485.2016 (2016). doi:10.1152/ajprenal.00485.2016

3. Letts, R. F. R., Rubin, D. M., Louw, R. H. & Hildebrandt, D. Glomerular protein separation as a mechanism for powering renal concentrating processes. *Med. Hypotheses* **85,** 120–123 (2015).

4. Moeller, H. B. & Fenton, R. A. Cell biology of vasopressin-regulated aquaporin-2 trafficking. *Pflugers Arch. Eur. J. Physiol.* **464,** 133–144 (2012).

5. Umenishi, F., Narikiyo, T., Vandewalle, A. & Schrier, R. W. cAMP regulates vasopressin-induced AQP2 expression via protein kinase A-independent pathway. *Biochim. Biophys. Acta - Biomembr.* **1758,** 1100–1105 (2006).

6. Kortenoeven, M. L. A. *et al.* In mpkCCD cells, long-term regulation of aquaporin-2 by vasopressin occurs independent of protein kinase A and CREB but may involve Epac. *Am. J. Physiol. Renal Physiol.* **302,** F1395–1401 (2012).

7. Bouley, R., Hasler, U., Lu, H. A. J., Nunes, P. & Brown, D. Bypassing Vasopressin Receptor Signaling Pathways in Nephrogenic Diabetes Insipidus. *Semin. Nephrol.* **28,** 266–278 (2008).

8. Bustamante, M. *et al.* Insulin potentiates AVP-induced AQP2 expression in cultured renal collecting duct principal cells. *Am. J. Physiol. Renal Physiol.* **288,** F334–344 (2005).

9. Hasler, U. *et al.* NF-kappaB modulates aquaporin-2 transcription in renal collecting duct principal cells. *J. Biol. Chem.* **283,** 28095–28105 (2008).

10. Hasler, U. Controlled aquaporin-2 expression in the hypertonic environment. *Am. J. Physiol. Cell Physiol.* **296,** C641–653 (2009).

11. Trott, O. & Olson, A. J. AutoDock Vina: improving the speed and accuracy of docking with a new scoring function, efficient optimization, and multithreading. *J. Comput. Chem.* **31,** 455–461 (2010).
